# Epigenetic re-expression of HIF-2α suppresses soft tissue sarcoma growth

**DOI:** 10.1038/ncomms10539

**Published:** 2016-02-03

**Authors:** Michael S. Nakazawa, T. S. Karin Eisinger-Mathason, Navid Sadri, Joshua D. Ochocki, Terence P. F. Gade, Ruchi K. Amin, M. Celeste Simon

**Affiliations:** 1 Abramson Family Cancer Research Institute, University of Pennsylvania, BRB II/III Room 456, 421 Curie Boulevard, Philadelphia, Pennsylvania 19104, USA; 2 Department of Cell and Developmental Biology, University of Pennsylvania, Philadelphia, Pennsylvania 19104, USA; 3 Department of Pathology and Laboratory Medicine, Perelman School of Medicine at the University of Pennsylvania, Philadelphia, Pennsylvania 19104, USA; 4 Department of Radiology, Perelman School of Medicine at the University of Pennsylvania, Philadelphia, Pennsylvania 19104, USA; 5 Howard Hughes Medical Institute, Philadelphia, Pennsylvania 19104, USA

## Abstract

In soft tissue sarcomas (STS), low intratumoural O_2_ (hypoxia) is a poor prognostic indicator. HIF-1α mediates key transcriptional responses to hypoxia, and promotes STS metastasis; however, the role of the related HIF-2α protein is unknown. Surprisingly, here we show that HIF-2α inhibits high-grade STS cell growth *in vivo*, as loss of HIF-2α promotes sarcoma proliferation and increases calcium and mTORC1 signalling in undifferentiated pleomorphic sarcoma and dedifferentiated liposarcoma. We find that most human STS have lower levels of *EPAS1* (the gene encoding HIF-2α) expression relative to normal tissue. Many cancers, including STS, contain altered epigenetics, and our findings define an epigenetic mechanism whereby *EPAS1* is silenced during sarcoma progression. The clinically approved HDAC inhibitor Vorinostat specifically increases HIF-2α, but not HIF-1α, accumulation in multiple STS subtypes. Vorinostat inhibits STS tumour growth, an effect ameliorated by HIF-2α deletion, implicating HIF-2α as a biomarker for Vorinostat efficacy in STS.

## Abstract (web summary)

Hypoxia is a common feature of soft tissue sarcomas, resulting in the activation of HIF-1α, which promotes metastasis. Here, Nakazawa *et al.* show that paradoxically HIF-2α is epigenetically silenced during the progression of multiple sarcoma subtypes, and when reexpressed, blocks tumour growth *in vivo*.

## Graphical abstract

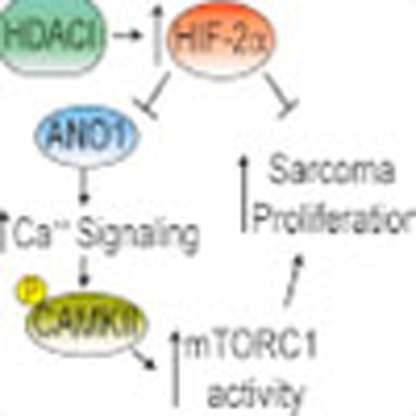

Soft tissue sarcomas (STS) are a diverse group of malignancies arising from mesenchymal tissues, currently classified into ∼50 distinct histological subtypes^1^. Each year, 12,000 new cases are diagnosed in the United States, and roughly 4,000 succumb to this disease^2^,^3^. While recent findings have defined molecular mechanisms underlying sarcomagenesis and disease progression, these cancers remain relatively understudied due to their varied clinical and pathological aetiologies, making effective treatment challenging^4^. Current therapeutic options for localized disease include surgical resection, frequently in combination with radiation therapy and chemotherapy. For metastatic or unresectable STS, cytotoxic chemotherapy remains the primary approach; however, response rates are only 10–25% (refs 5, 6). Therefore, it is critical to identify novel therapeutics, as well as biomarkers to predict their efficacy, to help improve patient outcomes.

Undifferentiated pleomorphic sarcoma (UPS), fibrosarcoma and dedifferentiated liposarcoma (DD-LPS) are undifferentiated high-grade sarcomas, which collectively represent up to 40% of newly diagnosed sarcomas in adults^7^. UPS is among the most aggressive STS subtypes in adults, with a five-year survival rate of only 24% in patients with metastatic disease^1^,^8^. Although UPS comprises 15% of newly diagnosed STS cases, its dedifferentiated phenotype suggests that it may represent a morphological end point for many other sarcomas^7^,^9^. Further characterization may therefore provide broader insights into other aggressive STS subtypes.

One prominent feature of STS, including UPS, are severely hypoxic regions, a phenotype associated with lower overall survival rates^10^,^11^,^12^. Cellular adaptation to hypoxic stress requires coordinated changes in gene expression, many of which are mediated by hypoxia-inducible factor (HIF)-1α and HIF-2α (13,14,15). Although HIF-1α and HIF-2α are stabilized under hypoxic conditions, extensive data indicate that many important HIF targets are controlled specifically by one isoform or the other^16^,^17^,^18^,^19^. Additionally, the impact of HIF-α isoform stabilization is context-dependent, as they have been demonstrated to promote or suppress tumour growth in different cancers^17^,^20^,^21^. Several HIF inhibitors have been developed for clinical intervention, and while certain compounds demonstrate isoform-specific inhibition^22^, many affect both HIF-1α and HIF-2α equally^23^,^24^. Thus, the role of both HIF-α subunits in specific tumour contexts must be characterized before using either pan or isoform-specific HIF-α drugs.

Whereas HIF-1α has recently been shown to promote metastasis in UPS and fibrosarcoma^25^, the role of HIF-2α in STS has not been established. In this study, using a genetically engineered UPS mouse model that faithfully recapitulates human disease^26^,^27^, as well as fibrosarcoma and liposarcoma xenografts, we found that HIF-2α expression surprisingly suppresses tumourigenesis. Loss of HIF-2α (encoded by the *EPAS1* gene) increased sarcoma tumour cell proliferation. Additionally, RNA-seq analysis indicated that *Anoctamin-1* (*ANO1*, *DOG1* and *TMEM16A*)^28^, encoding a calcium-activated chloride channel, was expressed at elevated levels in HIF-2α deficient autochthonous UPS tumours relative to controls. In turn, ANO1 overexpression coincided with elevated CAMKII and mTORC1 signalling in these tumours. mTORC1 senses nutrient availability, and regulates cellular growth, biosynthetic activity and ribosomal biogenesis^29^; as such, disregulation of this pathway occurs in a variety of cancer types, including sarcomas^30^. Decreased *EPAS1* messenger RNA (mRNA) expression (with no copy number variation) was detected in the majority of STS patient samples analysed, including UPS, fibrosarcoma and liposarcoma. These data suggest that *EPAS1* expression is suppressed by epigenetic mechanisms in multiple sarcoma subtypes. Altered epigenetics have been observed in many cancers, with dysregulation of the epigenome proposed as an important mechanism whereby tumours progress^31^. Of note, treatment with the chromatin modifying agent suberanilohydroxamic acid (SAHA, Sigma-Aldrich, St Louis, MO, USA; Vorinostat), a clinically approved histone deacetylase inhibitor (HDACi)^32^, significantly increased HIF-2α expression in several STS, and inhibited growth in a HIF-2α dependent manner.

## Results

### HIF-2α suppresses tumour growth in UPS

**Figure 1:**
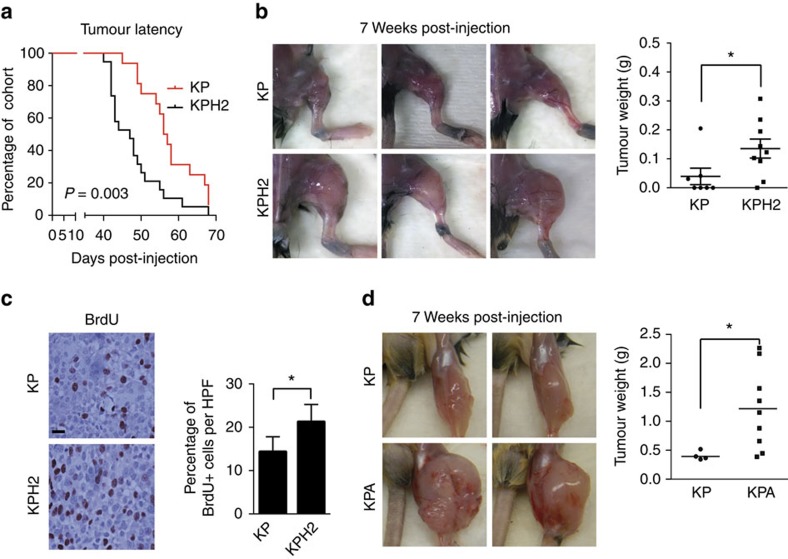
HIF-2α suppresses tumour growth in UPS. (**a**) Tumour latency of *LSL-Kras*^*G12D/+*^*; Trp53*^*fl/fl*^ (KP, *n*=16) and *LSL-Kras*^*G12D/+*^*; Trp53*^*fl/fl*^*; Epas1*^*fl/fl*^ (KPH2, *n=*19) mice, shown as days post-injection of Ad-Cre virus. The *P*-value was calculated using a log-rank (Mantel-Cox) test. (**b**) Left: a cohort of KP (*n=*7) and KPH2 (*n=*9) mice were killed 7 weeks post-Ad-Cre virus injection. Representative images of the hind limb where Ad-Cre was injected in each cohort are shown. Right: weight of KP and KPH2 tumours at 7 weeks post-Ad-Cre injection (grams; error bars are±s.e.m.). *P<0.05. (**c**) Immunohistochemical staining of BrdU in KP (*n=*5) and KPH2 (*n*=5) tumours. Left: images representative of KP and KPH2 cohort. Right: quantification of percentage of BrdU^+^ cells in KP and KPH2 tumours, over 10 high power fields (HPF) per sample (error bars are ±s.e.m.). *P<0.05. Scale bar, 25 μm. (**d**) Left: representative images of the hind limb of KP (*n*=5) and *LSL-Kras*^*G12D/+*^*; Trp53*^*fl/fl*^*; Arntfl*^*/fl*^ KPA (*n*=9) mice 7 weeks post-Ad-Cre virus injection. Right: weights of KP and KPA tumours 7 weeks post-Ad-Cre virus injection (grams; error bars are±s.e.m.). *P<0.05. *P*-values were calculated from a two-tailed Student's *t*-test for parts **b**–**d**.

To address the role of HIF-2α in STS, we crossed the previously described autochthonous ‘KP' (*LSL-Kras*^G12D/+^; *Trp53*^fl/fl^) UPS mouse strain^27^ with *Epas1*^fl/fl^ mice^33^ to generate ‘KPH2' (*LSL-Kras*^G12D/+^; *Trp53*^fl/fl^; *Epas1*^fl/fl^) animals. Hind limb UPS tumours are generated by injection of adenovirus expressing Cre-recombinase (Ad-Cre) into the gastrocnemius muscle. Of note, KPH2 mice formed UPS lesions at a significantly elevated rate compared with KP control animals, with 50% of KP and KPH2 strains exhibiting palpable tumours 56 and 47 days, respectively, after Ad-Cre injection (Fig. 1a). PCR analysis confirmed efficient *Epas1* deletion in KPH2 tumours (Supplementary Fig. 1a). Both KP and KPH2 samples displayed a similar heterogeneous, multinucleated appearance consistent with UPS and local muscle invasion was also observed, albeit more extensively in the KPH2 than KP tumours (Supplementary Fig. 1b). In agreement with prior reports^25^, KP and KPH2 tumours exhibit areas of localized hypoxia, as demonstrated by Hypoxyprobe staining (Supplementary Fig. 1c). Interestingly, KPH2 tumours were larger than KP controls, with significantly increased mass at 7 weeks post-Ad-Cre injection (Fig. 1b). KPH2 tumours were also more proliferative than KP, as demonstrated by BrdU uptake (Fig. 1c), while apoptotic rates were unchanged based on cleaved caspase-3 levels (Supplementary Fig. 1d)

Although HIF-1α and HIF-2α have unique transcriptional targets, they also regulate common genes in a coordinate or even opposing manner^16^,^17^,^18^. To rule out the possibility that the effects of HIF-2α deficiency *in vivo* were due to compensatory HIF-1α activity, we deleted *Arnt*, the obligate binding partner of both HIF-1α and HIF-2α. Compared with KP mice*, LSL-Kras*^G12D/+^; *Trp53*^fl/fl^; *Arnt*^fl/fl^ (KPA) animals generated larger lesions, phenocopying KPH2 tumour characteristics. Seven weeks following Ad-Cre injection, KPA tumours had significantly greater mass (Fig. 1d), indicating that the effects of HIF-2α deletion are not due to HIF-1α-mediated compensation. Together, these data indicate that HIF-2α suppresses UPS tumourigenesis, in contrast to HIF-1α which has no effect on primary tumour growth in this model^25^.

### HIF-2α loss promotes DD-LPS and fibrosarcoma growth

**Figure 2:**
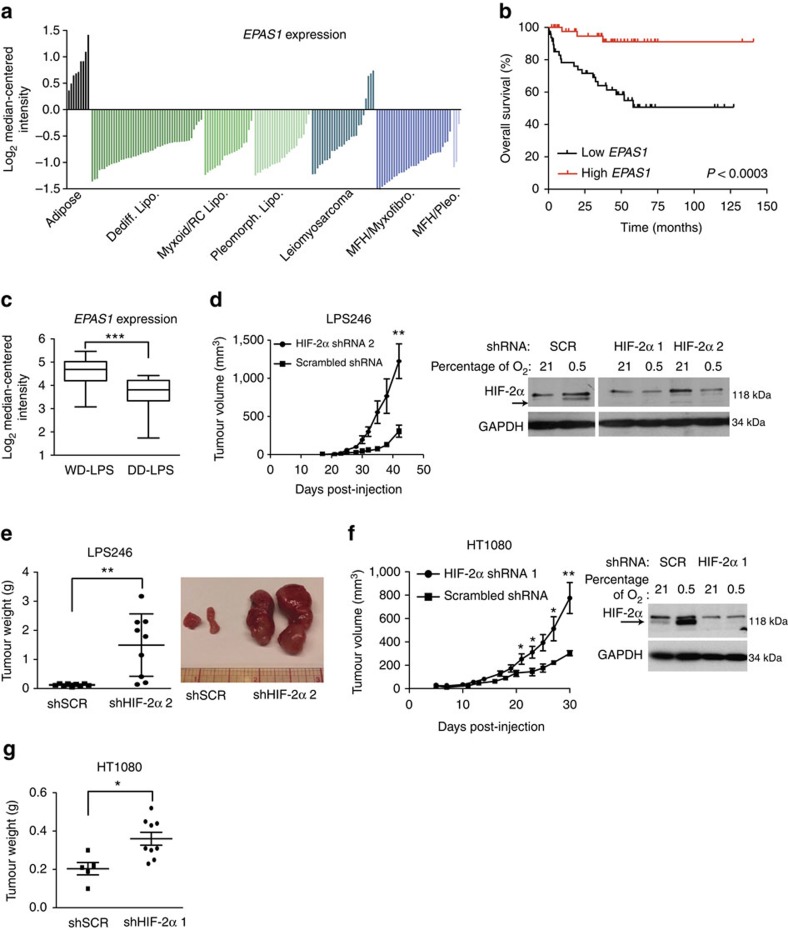
Loss of HIF-2α promotes liposarcoma and fibrosarcoma tumour growth *in vivo.* (**a**) *EPAS1* mRNA expression from Oncomine analysis of the Barretina *et al.* sarcoma patient samples data set^34^. Values are normalized to median-centered intensity, and shown on a log_2_ scale. Dediff. lipo., dedifferentiated liposarcoma; MFH/myxofibro., myxofibrosarcoma; MFH/pleo., UPS; myxoid/RC lipo., myxoid/round cell liposarcoma; pleomprh. lipo., pleomorphic liposarcoma. (**b**) Kaplan–Meier curve of overall survival of liposarcoma patients from the Gobble *et al.* data set^35^, segregated into the bottom 50% *EPAS1* expression (Low *EPAS1*, *n*=47) and top 50% *EPAS1* expression (High *EPAS1*, *n*=47). The *P*-value was calculated using a log-rank (Mantel–Cox) test. (**c**) *EPAS1* mRNA expression of well-differentiated liposarcoma (`WD-LPS', *n*=52) compared with dedifferentiated liposarcoma (`DD-LPS', *n*=20) patient samples from the Gobble *et al.* data set. Values are normalized to median-centered intensity, and shown on a log_2_ scale. ***P<0.001. (**d**) Left: tumour volume of LPS246 liposarcoma xenografts with scrambled (SCR; *n*=9) or HIF-2α shRNA (*n*=9). Right: immunoblot demonstrating HIF-2α knockdown with two independent HIF-2α shRNAs compared with SCR shRNA. **P<0.01. (**e**) Left: weights of LPS246 xenograft tumours with SCR shRNA (shSCR) or HIF-2α shRNA (shHIF-2α 2) at time of killing, measured in grams. **P<0.01. Right: image of representative LPS246 tumours with shSCR or HIF-2α shRNA (shHIF-2α 2). (**f**) Left: tumour volume of HT-1080 fibrosarcoma xenografts with SCR (*n*=5) or HIF-2α shRNA (HIF-2α 1; *n*=10). Right: immunoblot demonstrating HIF-2α knockdown with HIF-2α shRNA (HIF-2α 1) compared with SCR shRNA. **P<0.01. (**g**) Weights of HT-1080 xenograft tumours with shSCR and HIF-2α shRNA (shHIF-2α 1) at time of killing, measured in grams. *P<0.05. All error bars represent the mean±s.e.m. *P*-values for part **c**–**g** were calculated using a two-tailed Student's *t*-test.

To determine whether HIF-2α expression was decreased in other STS subtypes, we first queried publicly available microarrays of human STS using Oncomine. Analysis of a data set originally published by Barretina *et al.*^34^ showed that *EPAS1* mRNA expression was lower in fibrosarcoma, leiomyosarcoma, liposarcoma and UPS (previously named malignant fibrous histiocytoma) patient samples compared with normal adipose tissue (Fig. 2a). Additionally, liposarcoma and UPS tumours demonstrated decreased immunostaining for HIF-2α protein compared with normal artery and skeletal muscle tissue (Supplementary Fig. 2a). In a separate data set^35^, decreased *EPAS1* mRNA expression correlated with worse overall survival in liposarcoma patients (Fig. 2b). Of note, *EPAS1* levels were significantly lower in more aggressive liposarcoma subtypes, such as dedifferentiated and pleomorphic liposarcoma (Fig. 2c and Supplementary Fig. 2b), and in this data set, the bottom 50% *EPAS1* expressing cohort have a higher risk of death (relative risk of 6.67) compared with the top 50% *EPAS1* expressing cohort. Collectively, these analyses indicate *EPAS1* expression is decreased in multiple STS subtypes, and lower *EPAS1* levels correlate with poorer prognosis in a cohort of liposarcoma patients.

To test the functional effects of HIF-2α suppression in different STS subtypes, we inhibited HIF-2α using two independent shRNAs in LPS246 liposarcoma cells, which resulted in increased xenograft volume growth and mass (Fig. 2d,e, Supplementary Fig. 2c). Furthermore, HIF-2α depletion promoted growth of HT-1080 fibrosarcoma tumour xenografts *in vivo* (Fig. 2f,g, Supplementary Fig. 2d). Importantly, HIF-1α ablation did not affect tumour growth (Supplementary Fig. 2d), consistent with previous reports^25^. These results demonstrate that inhibition of HIF-2α *in vivo* accelerates growth of multiple STS, including UPS, fibrosarcoma and liposarcoma.

### Loss of HIF-2α increases mTORC1 signalling in STS

**Figure 3:**
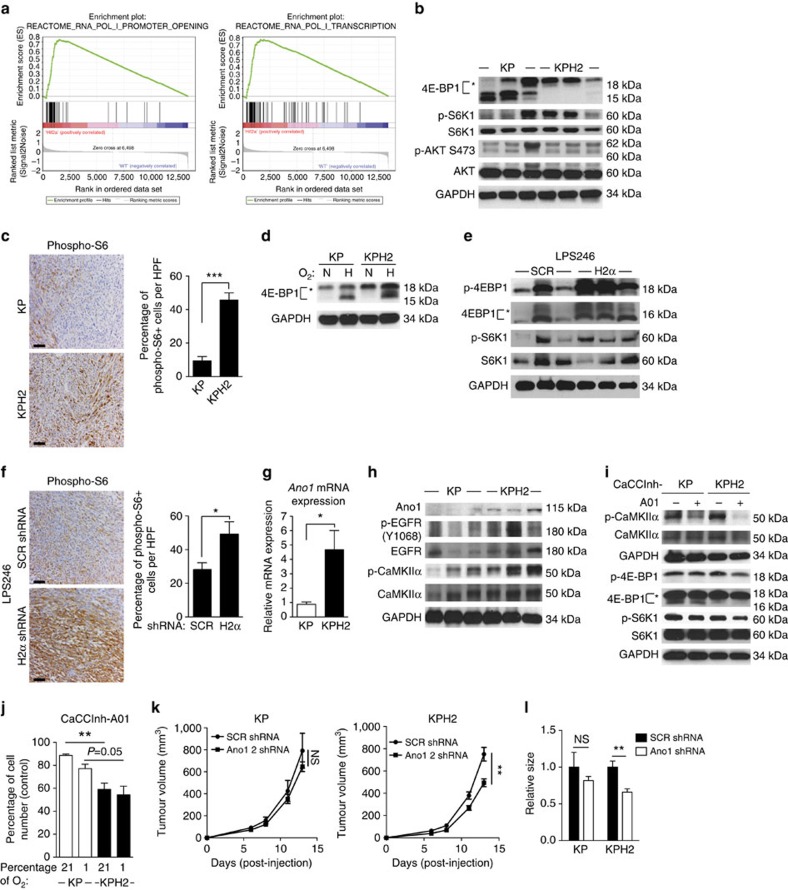
Loss of HIF-2α increases mTORC1 signalling in soft tissue sarcomas. (**a**) Gene set enrichment analysis (GSEA) comparing expression data of KP ‘WT' and KPH2 ‘Hif2a' autochthonous UPS tumours. (**b**) Immunoblot assessing mTORC1 and mTORC2 activity in KP and KPH2 tumours. Phosphorylated-(p) 4E-BP1 (indicated with an *) and S6K1 were used as mTORC1 readouts, and (p)-AKT was used as an mTORC2 readout. (**c**) Left: representative images of immunohistochemical staining of phosphorylated-S6 (phospho-S6) on KP (*n*=5) and KPH2 (*n*=5) tumours. Right: quantification of phospho-S6^+^ cells in KP and KPH2 tumours. 10 high-powered fields per tumour were quantified. ***P<0.001. (**d**) Immunoblot of 4E-BP1 in cell lines derived from KP and KPH2 tumours. Cells were subjected to 1% O_2_ for 16 h (H) or grown at 21% O_2_ (N). (**e**) Expression of 4E-BP1 and S6K1 phosphorylation in LPS246 xenografts with scrambled (SCR) or HIF-2α (H2α) shRNA. (**f**) Left: representative images of phospho-S6 immunohistochemical staining on LPS246 xenografts with SCR (*n*=5) or HIF-2α (H2α) shRNA (*n*=5; error bars are ±s.e.m.). 10 high-powered fields per tumour were quantified. *P<0.05. (**g**) Quantitative reverse trascriptase-PCR validation of *Ano1* mRNA expression in KP (*n*=4) and KPH2 (*n*=3) tumours used for RNA-seq *P<0.05. (**h**) Immunoblot of ANO1 and downstream targets p-EGFR (Y1068) and p-CaMKIIα (T268) in KP and KPH2 autochthonous tumours. (**i**) KP and KPH2 cells were serum starved for 24 h, then replete media with DMSO or CaCCInh-A01 (10 μM) was added for 6 h to the cells. Lysates were immunoblotted for p-CAMKIIα and mTORC1 readouts p-4E-BP1 and p-S6K1. (**j**) ANO1 inhibitor's effect on KP and KPH2 cell growth. Cells were treated with DMSO or CaCCInh-A01 (10 μM) for 3 days in 21% or 1% O_2_ conditions. Shown is the percentage of cells counted in the CaCCInh-A01 treated versus the respective DMSO-treated control, with each bar representing three biological triplicates. **P<0.01. (**k**) Left: tumour volume of KP-derived UPS allografts expressing SCR (*n*=5) or *Ano1* shRNA (Ano1 2; *n*=5). Right: tumour volume of KPH2-derived UPS allografts expressing SCR (*n*=5) or *Ano1* shRNA (*n*=5). **P<0.01. (**l**) Relative average size of KP and KPH2 tumours infected with SCR or *Ano1* shRNA. The SCR shRNA average volume was normalized to 1.0 for both KP and KPH2 cohorts. **P<0.01. All error bars represent the mean±s.e.m. All *P-*values were calculated using a two-tailed Student's *t*-test.

To define mechanism(s) whereby HIF-2α opposes sarcomagenesis using an unbiased approach, we performed RNA-seq analysis of KP (*n*=3) and KPH2 (*n*=4) tumours. By principle component analysis, KP and KPH2 tumours segregated into distinct populations (Supplementary Fig. 3a). Gene set enrichment analysis of differentially expressed genes revealed a strong enrichment for those regulating RNA polymerase I promoter opening and transcription in KPH2 compared with KP samples (Fig. 3a and Supplementary Fig. 3b), suggesting increased ribosome biogenesis in the KPH2 tumours. As c-MYC and mTORC1 signalling promote proliferation and ribosome biogenesis in cancers^29^,^36^, we assessed whether either pathway was more active in KPH2 tumours compared with KP controls. KPH2 tumours displayed increased c-Myc protein levels on average, but only modest changes in the expression of several canonical c-MYC target genes (*Myc*, *Ccnd1*, *Ccdn2*, *Mcm5* and *Cdkn1a*; Supplementary Fig. 3c) were observed (for more discussion on this matter, see below). Additionally, Ingenuity Pathway Analysis (IPA) did not predict c-MYC activation in KPH2 tumours. In contrast, IPA predicted increased mTOR activity based on differentially expressed mTOR targets between KP and KPH2 tumours (Supplementary Fig. 3d). The canonical mTORC2 target, phosphorylated AKT at serine 473, was not significantly different between KP and KPH2 tumours (Fig. 3b)^37^. However, using a Fischer's exact test, we determined that KPH2 tumours had a statistically significant mTORC1 target gene signature, for targets predicted to be both elevated (*P*=0.014) and suppressed (*P*=0.010) by mTORC1 (ref. 38). Canonical mTORC1 signalling outputs, phosphorylated 4E-BP1 and S6K1 (ref. 39), were also assessed in KP and KPH2 tumours, and elevated 4E-BP1 phosphorylation was observed in KPH2 tumours compared with KP (Fig. 3b). We perceived variable increases in S6K1 phosphorylation in whole KP and -KPH2 UPS lysates, indicating that mTORC1 activity may vary in tumour subdomains. Therefore, we examined phosphorylated-S6 staining in tumour parenchyma by IHC, and noted significantly expanded areas of phosphorylated-S6 in KPH2 (*n*=5) compared with KP (*n*=5) sections (Fig. 3c). Phosphorylated 4E-BP1 levels were elevated in KPH2 tumour-derived cells as compared with KP tumour-derived cells (Supplementary Fig. 3e), with the expected increase in unphosphorylated 4E-BP1 under hypoxic growth conditions (Fig. 3d). Consistent with this finding, proliferation was also increased in KPH2 cells (Supplementary Fig. 3f). Similar increases in phosphorylated 4E-BP1, S6K1 and S6 were apparent in HIF-2α knockdown LPS246 xenografts (Fig. 3e,f). Although some heterogeneity exists in tumours expressing or lacking HIF-2α, taken together, these data indicate that mTORC1 activity is increased in HIF-2α deficient sarcomas.

To elucidate the mechanisms whereby HIF-2α alters mTORC1 activity in UPS, we examined the top differentially expressed genes base on RNA-seq data. Several targets identified were related to skeletal muscle and extracellular matrix (for example, *Lama5*, *Col18a1* and *Dmp1*), consistent with KPH2 showing more local invasion into the adjacent skeletal musculature (Supplementary Figs 1b and 4a). However, *Ano1* (encoding *TMEM16A*, *Anoctamin-1* and *DOG1*) transcripts were significantly increased in KPH2 tumours relative to controls (Fig. 3g). ANO1, a calcium-activated chloride channel^28^, is overexpressed in a variety of cancers, including gastrointestinal stromal tumours, breast cancer, and head and neck cancers^40^,^41^,^42^. KPH2 tumours also exhibited elevated ANO1 protein levels compared with KP tumours (Fig. 3h), although the available antibody exhibited relatively weak immunoreactivity with both KP and KPH2 tumour lysates.

ANO1 has previously been reported to promote both EGFR and CaMKIIα signalling in breast cancer cell lines^42^. While we failed to observe a consistent pattern of increased EGFR phosphorylation in KPH2 tumours (Fig. 3h), these tumours expressed higher levels of phosphorylated CaMKIIα. These data are consistent with IPA analyses, which predicted increased calcium signalling in KPH2 tumours (Supplementary Fig. 4b). Since intracellular calcium and CaMKIIα have been shown to enhance mTORC1 signalling^43^,^44^, we hypothesized that increased mTORC1 activity in KPH2 tumours was mediated through ANO1 and CAMKII signalling. To test this, we serum starved KP and KPH2 tumour-derived cells for 24 h, and then treated with replete media plus dimethyl sulphoxide (DMSO) or CaCCinh-A01, a small molecule inhibitor of ANO1 activity^42^. KPH2 cells maintained slightly higher levels of ANO1 compared with KP *in vitro* (Supplementary Fig. 4c), although this phenotype is more striking in tumours as they were cultured under normoxic conditions. As expected, CaCCinh-A01 treatment reduced phosphorylated CaMKIIα levels more effectively in KPH2 compared with KP cells (Fig. 3i). Moreover, CaCCinh-A01 diminished mTORC1 activation (Fig. 3i), consistent with our findings that ANO1 is more highly expressed in KPH2 cells. In addition, CaCCinh-A01 treatment decreased cell proliferation more potently in KPH2 than KP cells after 3 days of treatment (Fig. 3j). To determine if ANO1 inhibition diminished HIF-2α deficient sarcoma growth *in vivo*, *Ano1* targeted shRNAs were introduced into KP and KPH2-derived UPS cells (Supplementary Fig. 4d,e) to generate allografts. Similar to our *in vitro* data, inhibition of ANO1 reduced tumour growth in KPH2 but not KP allografts (Fig. 3k,l). Collectively, these data suggest that loss of HIF-2α increases ANO1 accumulation, activating CaMKII and mTORC1 signalling in UPS tumours, enhancing their growth (see below for further discussion).

### HDAC inhibition increases *EPAS1* expression in UPS

**Figure 4:**
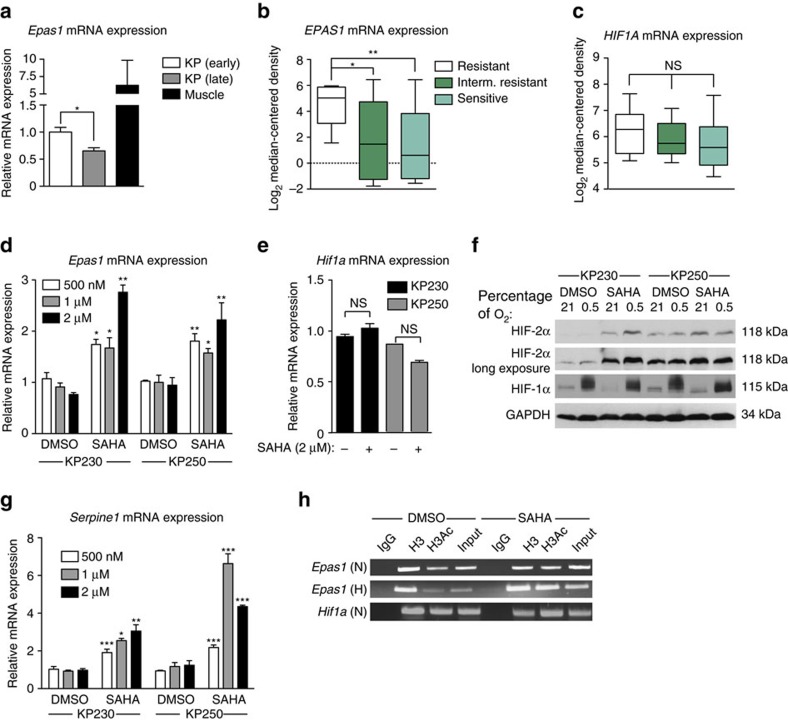
HDAC inhibition increases HIF-2α expression in UPS. (**a**) *Epas1* mRNA expression of tumours isolated from KP mice 7 weeks after Ad-Cre injection (early; *n*=4), 8.5 weeks after Ad-Cre injection (late; *n*=4), and whole-mouse gastrocnemius muscle from uninjected muscle (*n*=3). (**b**) *EPAS1* mRNA expression of sarcoma cell lines classified as SAHA resistant (9 cell lines), intermediate resistant (Interm. Resistant; 14 cell lines) and sensitive (12 cell lines) from the Oncomine analysis of Garnett *et al.* data set^45^. Values are normalized to median-centered intensity, and shown on a log_2_ scale. *P<0.05, **P<0.01. (**c**) *HIF1A* mRNA expression from the Garnett *et al.* data set (error bars are±s.e.m.). SAHA resistant, 9 cell lines; Intermediate resistant (Interm. Resistant), 13 cell lines; Sensitive, 12 cell lines. (**d**) *Epas1* mRNA expression of KP230 and KP250 mouse UPS cell lines treated with DMSO or SAHA at the indicated drug concentrations. Each bar represents three independent experiments performed in triplicate. *P<0.05, **P<0.01. (**e**) *Hif1a* mRNA expression of KP230 and KP250 cells treated with DMSO or SAHA (2 μM). Each bar represents three independent experiments performed in triplicate. (**f**) Immunoblot for HIF-1α and HIF-2α protein in KP230 and KP250 cells treated with DMSO or SAHA (2 μM), grown under 21% O_2_ or 0.5% O_2_ conditions. (**g**) *Serpine1* mRNA expression of KP230 and KP250 cells treated with DMSO or SAHA at the indicated drug concentrations. Each bar represents three independent experiments performed in triplicate. *P<0.05, **P<0.01, ***P<0.001. (**h**) ChIP of KP250 cell treated with DMSO or SAHA (2 μM), grown under 21% O_2_ (N) or 0.5% O_2_ (H) conditions. ChIP primers used amplified the 500-bp fragment upstream of the *Epas1* and *Hif1a* TSSs. Histone H3 and acetylated histone H3 specific (H3Ac) antibodies were used for ChIP, with IgG as negative control. Input was diluted 1:100. Results are representative of two independent experiments. All data shown are the mean±s.e.m. All *P*-values were calculated using a two-tailed Student's *t*-test.

Having determined that HIF-2α exhibits a tumour suppressive role in UPS and other STS, we examined possible mechanisms whereby HIF-2α is silenced in these lesions. Initially, we analysed *Epas1* expression over time in the KP tumour model. One cohort of animals was euthanized 7 weeks post-Ad-Cre injection (‘KP early', *n*=7), and another at 8.5 weeks post-Ad-Cre injection (‘KP late', *n*=9). *Epas1* mRNA levels in the KP late cohort were significantly diminished compared with KP early, with *Epas1* mRNA of mouse skeletal muscle shown as a comparison (Fig. 4a). Therefore, we concluded that *Epas1* expression is lost progressively over the course of sarcomagenesis. Analysis of TCGA sarcoma patient samples revealed that 25% of all human sarcomas have lost at least one copy of *EPAS1*, while 67% remain diploid (Supplementary Fig. 5a). As *EPAS1* mRNA levels are consistently diminished in several STS subtypes (Fig. 2a), we hypothesized that *EPAS1* is epigenetically silenced in a significant fraction. Using a publically available data set of human sarcoma cell lines treated with various cancer therapeutics, we identified a significant correlation between *EPAS1* mRNA abundance and sensitivity to the HDACi Vorinostat, also known as SAHA (Fig. 4b, Supplementary Fig. 5b)^45^. In contrast, no such correlation existed between *HIF1A* expression and SAHA sensitivity in the same cell lines (Fig. 4c). We also analysed DNA methylation along the *EPAS1* locus in the TCGA sarcoma data set, and found no consistent methylation changes in the samples (Supplementary Table 1). Together, these results suggest that HDACs specifically suppress *EPAS1* in sarcoma, and that re-expressing *EPAS1* could be a mechanism underlying HDACi's inhibition of sarcoma growth.

To test whether HDACi could activate *EPAS1* expression in sarcoma, two independent mouse UPS cell lines, derived from KP tumours (KP230 and KP250), were treated with multiple SAHA concentrations (500 nM–2 μM) *in vitro*. SAHA exposure increased *Epas1* mRNA levels (Fig. 4d), an effect also observed in HT-1080 cells (Supplementary Fig. 5c). Importantly, at the highest dose tested (2 μM), SAHA treatment had no effect on *HIF1A* mRNA in UPS or HT-1080 cell lines (Fig. 4e and Supplementary Fig. 5c). Consistent with these findings, 2 μM SAHA also increased HIF-2α protein levels in both KP230 and KP250 cells, while HIF-1α protein was unchanged (Fig. 4f). SAHA treatment also resulted in elevated mRNA levels of *Serpine1, a* HIF-2α target^46^, at all concentrations tested (Fig. 4g). We also investigated whether altering DNA methylation would impact *EPAS1* expression, given that DNA methylation regulates *EPAS1* in renal cell carcinoma^47^. However, treatment with 5-azacytidine, a DNA methyltransferase inhibitor^48^, and 3-Deazaneplanocin A (Cayman Chemicals, Ann Arbor, MI, USA), a EZH2 histone methyltransferase inhibitor, failed to significantly increase *Epas1* mRNA levels in UPS cell lines (Supplementary Fig. 5d). To rule out off-target effects of SAHA on *EPAS1*, we treated KP230, KP250 and HT-1080 cells with an independent class I/II HDACi, Trichostatin A (Supplementary Fig. 5e). Trichostatin A increased *Epas1* and *Serpine1* mRNA levels, but had no effect on *Hif1a* mRNA expression. However, treatment with nicotinamide, a class III HDACs/sirtuins inhibitor^49^, did not change *Epas1* or *Hif1a* mRNA levels in the same cell lines (Supplementary Fig. 5f). Finally, chromatin immunoprecipitation (ChIP) was performed to verify that SAHA exposure increased the abundance of acetylated histones at the *Epas1* locus. Using available ChIP-seq data from the UCSC genome browser of mouse liver and heart tissue, primers targeting the region 500 bp upstream of the *Epas1* transcription start site (TSS) were designed. ChIP-seq/RNA-seq analyses of *EPAS1* expressing tissues indicated this region is rich in histone acetylation. Treatment with 2 μM SAHA increased levels of acetylated histone H3 near the *Epas1* TSS compared with DMSO control under normoxic (21% O_2_) and hypoxic (0.5% O_2_) conditions (Fig. 4h). Conversely, no increases in acetylated histone H3 at 500 bp upstream of the *Hif1a* TSS were detected. In aggregate, these results indicate that HIF-2α expression is lost during UPS disease progression, and epigenetic therapeutics like SAHA specifically elevate HIF-2α, but not HIF-1α, levels.

### SAHA slows UPS growth in a HIF-2α dependent manner

**Figure 5:**
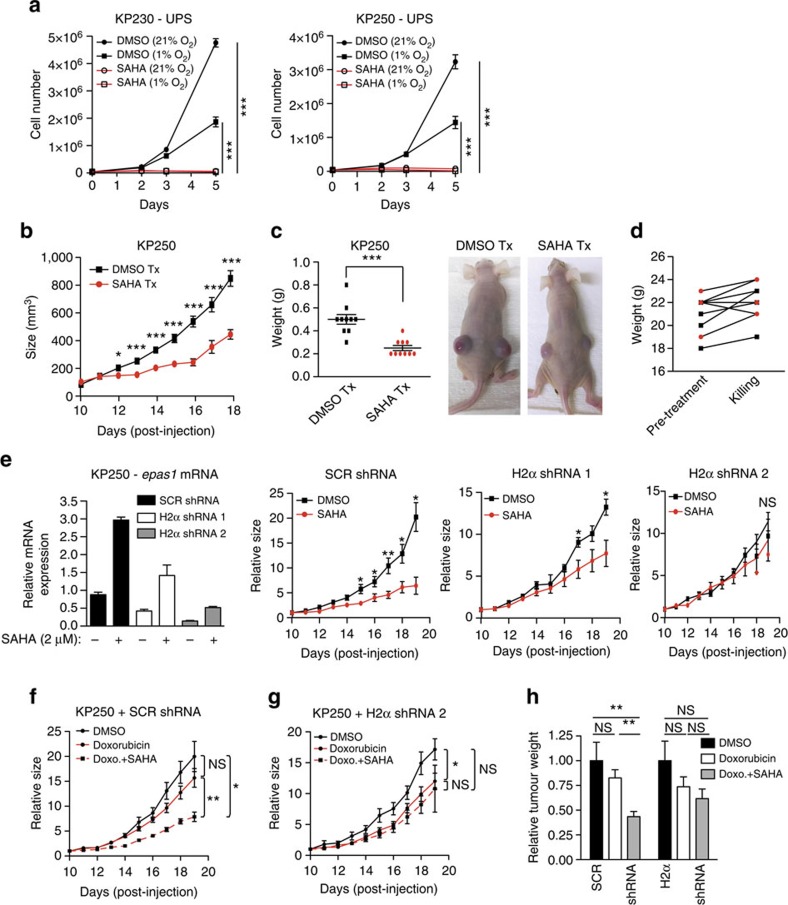
SAHA inhibition of UPS allograft growthis dependent on HIF-2α re-expression. (**a**) Proliferation of KP230 and KP250 cells treated with DMSO or SAHA (2 μM) under 21% O_2_ or 1% O_2_. Drug treatment began on day 1. Each line represents three independent experiments performed in duplicate. ***P<0.001. (**b**) Tumour size of subcutaneous (s.c.) KP250 allografts in DMSO (*n*=10) or SAHA (50 mg kg^−1^ per day)-treated mice (*n*=10). Mice were treated once tumours reached ∼100 mm^3^. *P<0.05, ***P<0.001. (**c**) Tumour weights from DMSO- and SAHA-treated mice. Images are representative of tumours from the DMSO and SAHA cohorts. ***P<0.001. (**d**) Weights of mice pre-treatment and at time of killing. Black squares, DMSO-treated mice; red circles, SAHA-treated mice. (**e**) Left: *Epas1* mRNA expression of KP250 cells harbouring scrambled (SCR) shRNA, and two independent HIF-2α (H2α) shRNAs, treated with DMSO or SAHA (2 μM) *in vitro*. Bars represent two independent experiments performed in triplicate. Right: Relative size increases of s.c. KP250 allografts with SCR shRNA, H2α 1 shRNA and H2α 2 shRNA-treated with DMSO or SAHA (50 mg kg^−1^ per day). Mice were treated 10 days post-injection of tumour cells. For each shRNA, 7 mice were treated DMSO and 7 mice were treated with SAHA. *P<0.05, **P<0.01. (**f**) Relative tumour size of s.c. KP250+SCR shRNA allografts treated with DMSO, doxorubicin or doxorubicin and SAHA. Treatment began 10 days post-injection of tumour cells. Doxorubicin was administered at 5 mg kg^−1^ per week, and SAHA was administered at 50 mg kg^−1^ per day. *P<0.05, **P<0.01. (**g**) Comparative tumour size of KP250+HIF-2α shRNA 2 (H2α) allografts treated with DMSO, doxorubicin or doxorubicin and SAHA. *P<0.05. (**h**) Relative tumour weights of KP250 allografts with SCR or H2α shRNA. Sizes of doxorubicin and doxorubicin with SAHA treatment are normalized to their respective DMSO control. **P<0.01. All data shown are the mean±s.e.m. All *P-*values were calculated using a two-tailed Student's *t*-test.

Findings described above suggest that SAHA treatment may suppress sarcoma cell proliferation and tumour growth. We first tested this hypothesis by investigating SAHA's ability to inhibit UPS, HT-1080 and LPS246 sarcoma cell proliferation *in vitro*. Of note, SAHA treatment significantly decreased cell growth under both normoxia and hypoxia (1% O_2_; Fig. 5a, Supplementary Fig. 6a,b).

Next, we evaluated SAHA's therapeutic efficacy against UPS allografts *in vivo,* and found that SAHA administration (50 mg kg^−1^ per day) significantly reduced tumour growth (Fig. 5b), and final tumour weights (Fig. 5c). Importantly, no obvious adverse effects of treatment were observed, and the mice maintained weight similar to DMSO-treated controls (Fig. 5d). Surprisingly, *Epas1* mRNA levels were only slightly elevated in the SAHA-treated tumours compared with controls, although there was no change in *Hif1a* levels, as expected (Supplementary Fig. 6c). We suspected that KP tumour cells developed resistance to SAHA, and its effect on *Epas1* re-expression, over the extended course of the experiment (8 days). Consistent with this hypothesis, tumours from UPS-bearing mice treated with SAHA for only 4 days displayed significantly increased *Epas1* mRNA levels (Supplementary Fig. 6d). As SAHA can affect multiple cellular targets and processes^50^, we assessed the specific role of HIF-2α in SAHA-mediated anti-tumour effects. Two independent shRNAs, one producing a partial inhibition (H2α shRNA 1), and a second more effective shRNA (H2α shRNA 2), were used to deplete HIF-2α in KP250 cells (Fig. 5e, Supplementary Fig. 6e). Both HIF-2α shRNAs inhibited SAHA-induced *Epas1* and *Serpine1* mRNA re-expression in UPS cells. Mice bearing *in vivo* UPS allografts expressing control or HIF-2α shRNAs were then treated with DMSO or SAHA (50 mg kg^−1^ per day) once tumours had reached 100 mm^3^ (10 days post-implantation). SAHA significantly slowed the growth of control tumours, while HIF-2α inhibition abrogated this effect in a dose-dependent manner (Fig. 5e). Conversely, overexpression of HIF-2α in HT-1080 fibrosarcoma cells inhibited cell proliferation *in vitro* under hypoxic conditions (Supplementary Fig. 6f,g). When treated with SAHA, we observed a further increase in HIF-2α protein in empty vector and HIF-2α overexpressing cells, and a further decrease in cell proliferation likely due to increased expression of the endogenous HIF-2α mRNA (Supplementary Fig. 6g,h).

The standard chemotherapeutic approach for unresectable STS is doxorubicin^5^. To demonstrate that SAHA treatment could be safely and effectively incorporated into the clinical setting, we investigated the efficacy of combining doxorubicin and SAHA. Although combination therapy reduced UPS allograft growth *in vivo* compared with DMSO or doxorubicin alone (Fig. 5f), this inhibition was similar to SAHA treatment alone (Fig. 5e). Moreover, HIF-2α inhibition in these allografts fully abrogated this effect. (Fig. 5g,h). Collectively, these data strongly suggest that SAHA limits UPS proliferation *in vitro* and *in vivo*, in a HIF-2α-dependent manner.

**Figure 6:**
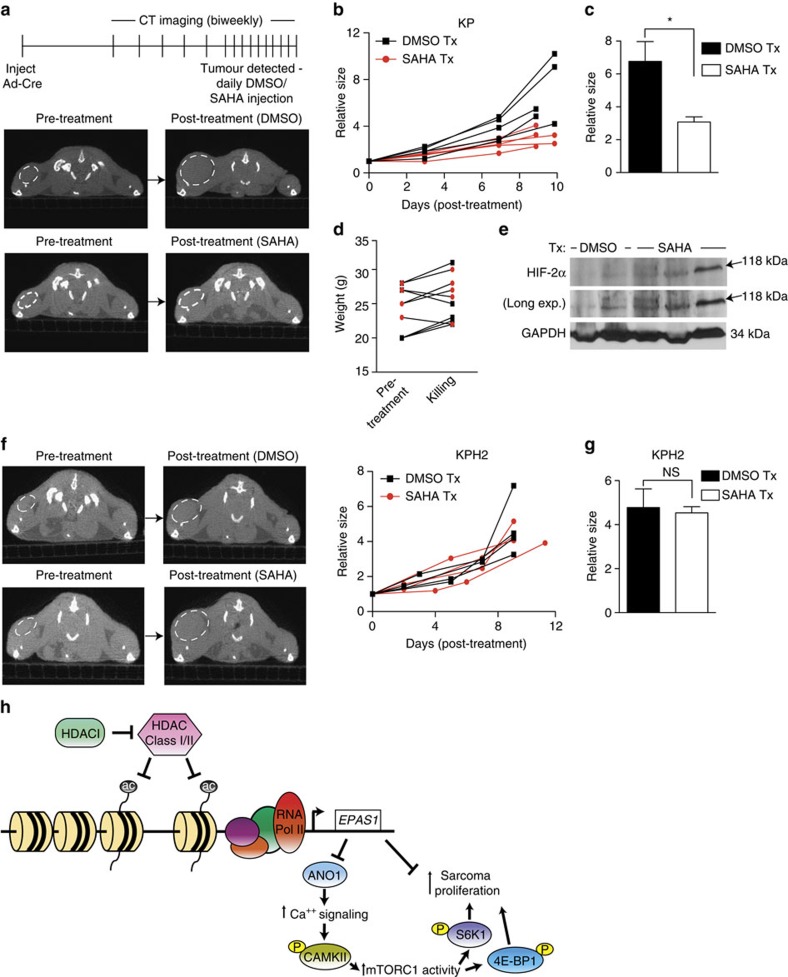
HIF-2α expression is required for SAHA's efficacy in an autochthonous UPS model. (**a**) Top: schematic of tracking autochthonous KP tumour growth in DMSO- and SAHA-treated mice. After Ad-Cre injection, mice were imaged bi-weekly until tumours were detectable and measured ∼50–100 mm^3^. Mice were randomized to DMSO control (*n*=5) or SAHA (*n*=5; 50 mg kg^−1^ per day) treatments, and tumour growth was followed by bi-weekly CT scans. Bottom: representative axial CT images of KP mice pre- and post-treatment with DMSO or SAHA. Dashed white line demarcates the tumour boundary. (**b**) Relative sizes of individual tumours from KP mice receiving DMSO or SAHA treatment. (**c**) Comparison of the relative sizes of all DMSO- and SAHA-treated KP tumours at the time of killing (error bars are±s.e.m.). *P<0.05. *P-*values were calculated from a two-tailed Student's *t*-test. (**d**) Weights of KP mice pre-treatment and at time of killing (error bars are±s.e.m.). Black squares, DMSO-treated mice; red circles, SAHA-treated mice. (**e**) Immunoblot of HIF-2α protein in KP autochthonous tumours from DMSO- and SAHA-treated mice. GAPDH served as loading control. (**f**) Left: representative transverse CT images of KPH2 mice pre- and post-treatment with DMSO or SAHA. Mice were treated and imaged as described in **a**. Right: relative sizes of individual tumours from KPH2 mice receiving DMSO (*n*=4) or SAHA (*n*=5) treatment. (**g**) Comparison of the relative sizes of all DMSO- and SAHA-treated KPH2 tumours at the time of killing (error bars are ±s.e.m.). (**h**) Model of *EPAS1* regulation in the STS examined. *EPAS1* expression is epigenetically downregulated through class I/II HDACs. Loss of HIF-2α increases ANO1 and calcium signalling, which subsequently increases mTORC1 activity in tumours and promotes sarcoma proliferation. HIF-2α loss may also increase sarcoma growth independent of this pathway.

We next evaluated the efficacy of SAHA in an autochthonous STS tumour model, using KP mice. After initiating tumours with Ad-Cre injection, we performed bi-weekly CT scans of the animals' lower limbs. When tumours reached 50–100 mm^3^, animals were treated with DMSO or SAHA (50 mg kg^−1^) daily (Fig. 6a). SAHA decreased relative tumour growth over time, compared with controls (Fig. 6b), resulting in significantly reduced relative tumour size by day 10. (Fig. 6c). Total animal weight did not change due to SAHA treatment (Fig. 6d). Importantly, HIF-2α protein levels increased in SAHA-treated tumours compared with controls (Fig. 6e). SAHA also decreased KP tumour cell proliferation, as indicated by reduced Ki67 staining (Supplementary Fig. 7a) with no concomitant change in apoptosis (Supplementary Fig. 7b). Importantly, SAHA's effects on tumour growth (Fig. 6f,g) and proliferation (Supplementary Fig. 7c,d) were completely abrogated in KPH2 tumours, underscoring the importance of HIF-2α in SAHA-mediated anti-tumour effects.

## Discussion

Current chemotherapeutic approaches for the treatment of unresectable and metastatic STS have low-response rates^5^,^6^. One major obstacle to developing better treatment regimens has been the myriad of unique subtypes with distinct genetic alterations^3^,^4^, coupled with the relatively low incidence of these malignancies. Until recently, clinical trials often combine patients with diverse STS histologic subtypes, which are induced by heterogeneous genetic alterations potentially from multiple cells of origin, into a single study. Our growing understanding of context specificity in tumours, particularly in sarcomas, has shown that this approach has many drawbacks. Newer targeted therapies are increasingly being investigated, not only in a sub-type specific manner, but also based on the molecular underpinnings of the tumours^51^. It will be very beneficial to identify therapeutic biomarkers that span multiple subtypes and determine treatment strategies based on gene expression changes that are linked to drug sensitivity. Identification of biomarkers that predict drug response should allow clinicians to better select patient populations most likely to respond to treatment^4^. Although these biomarkers are scarce in STS, we report here that HIF-2α expression levels may be particularly effective for this purpose.

Clinical data have identified intratumoural hypoxia and HIF-1α as one of the most important prognostic factors in metastatic potential of STS^52^,^53^. Consistent with this finding, we have shown previously that HIF-1α enhances tumour metastasis through modifications of the collagen network in UPS and fibrosarcoma^25^. Conversely, we demonstrate here that HIF-2α expression opposes UPS, fibrosarcoma and liposarcoma growth *in vivo*, decreasing tumour cell proliferation and inhibiting mTORC1 activity. Although tumours exhibit significant heterogeneity in our *in vivo* models, both within a given sample and between samples, we saw significant enrichment of mTORC1 activation. Importantly, further work is necessary to determine if this mechanism is shared with other STS subtypes not examined here. It is noteworthy that low HIF-2α expression is correlated with lower overall survival rate in a cohort of liposarcoma patients^35^. Additionally, loss of HIF-2α did not alter metastatic potential in UPS *in vitro* or *in vivo*. Although mTORC2 signalling has been shown to promote HIF-2α accumulation in cancers like renal cell carcinoma^54^, our results suggest that HIF-2α inhibits mTORC1 signalling in at least the high-grade STS subtypes examined. This context dependency of HIFs and mTOR signalling in cancer warrants further study. For UPS tumours, loss of HIF-2α increases ANO1, promoting CaMKII and mTORC1 activity (Fig. 6h). Interestingly, a previous study demonstrated that HIF-1α inhibition increased *ANO1* mRNA expression in renal cysts^55^, yet we are the first to connect HIF-2α loss with increased ANO1 in UPS. However, HIF-2α may also inhibit sarcoma growth in an ANO1 and mTORC1-independent fashion (Fig. 6h). Given the multitude of targets and biological processes HIF-2α controls, the mechanism whereby HIF-2α inhibition increases ANO1 levels remains to be elucidated. Importantly, ChIP-seq data assembled in the UCSC genome browser show Myc/Max binding near the promoter region of *ANO1* in breast and leukaemia cells. Thus, one possible explanation for our findings is that Myc, whose level is elevated in KPH2 tumours (Supplementary Fig. 3c), binds to the *Ano1* promoter and increases *Ano1* expression. Myc stimulation (or repression) of individual target genes can be modest^56^, and additional factors are required to regulate many Myc driven targets^36^. While the canonical Myc target genes we examined were slightly elevated in KPH2 tumours (or decreased in the case of *Cdkn1a*), *Ano1* expression was significantly increased, suggesting other input(s) are likely influencing its transcription. Clarifying the specific mechanism of HIF-2α mediated ANO1 regulation will be an important topic of future studies.

Observations that HIF-1α and HIF-2α can play different and often opposing roles in various malignancies is important as HIF inhibitors are systematically being developed as cancer therapies^16^. For example, HIF-2α in clear-cell renal cell carcinoma promotes tumourigenesis^17^,^57^, whereas it suppresses tumourigenesis in hepatocellular carcinoma and non-small cell lung adenocarcinoma^20^,^21^. Although HIF-α subunit specific inhibitors are currently under investigation^22^, pan-HIFα inhibitors are also being assessed^23^,^24^. Our results with ARNT knockout KP mice caution against the use of pan-HIFα inhibitors for the treatment of STS and potentially for other tumours as well.

Given that HIF-2α expression opposes STS tumourigenesis, we sought a class of compounds that could induce re-expression of HIF-2α in sarcoma cells, and found that the FDA-approved HDACi SAHA (Vorinostat) reactivates *EPAS1* expression in STS cells, inhibiting sarcoma progression (Fig. 6h). HDACi have been assessed *in vitro* and *in vivo* pre-clinically for the treatment of a variety of malignancies, including specific sarcoma subtypes such as malignant peripheral nerve sheath tumours^58^ and synovial sarcoma, with some early success^59^,^60^,^61^. Clinical trials testing chemotherapy with HDACi in STS are still ongoing, as well as newer generations of HDACi, such as abexinostat, with potentially improved anti-tumour effects and pharmokinetics^62^. To our knowledge this study is the first to show HDACi treatment reduces growth of autochthonous UPS tumours *in vivo*, and demonstrate that HIF-2α re-expression is required for SAHA's anti-proliferative effects in the context of UPS.

Importantly, combination of standard doxorubicin, a first-line chemotherapeutic against STS, with vorniostat (or newer generation HDACi such as abexinostat) could be a suitable treatment strategy that has exciting clinical potential. These results suggest that HDACi therapies will be most effective against sarcomas that have epigenetically suppressed HIF-2α expression, and not genomic deletion of the locus. HIF-2α may therefore serve as an important biomarker for clinicians designing future clinical trials with HDAC inhibitors in sarcoma patients.

## Methods

### Cell culture

HT-1080 (fibrosarcoma) and HEK-293T cell lines were purchased from ATCC (Manassas, VA, USA). KP210, KP230 and KP250 cell lines were derived from UPS mouse tumours^25^. These high-grade neoplasms showed myofibroblastic differentiation and atypical nuclei^27^, and by expression profiling matched human MFH/UPS (ref. 26). LPS246 was established from a primary dedifferentiated liposarcoma sample (confirmed with MDM2 FISH analysis), and was provided by Dr Dina Lev (Core Facilities, MD Anderson Cancer Center, Houston, TX)^63^. All cell lines were confirmed to be negative for mycoplasma contamination.

### Drug treatment *in vitro* and lentiviral transduction

For *in vitro* drug studies, SAHA and Trichostatin A (Sigma-Aldrich) were dissolved in DMSO, and diluted in media to final concentrations of 500 nM–2 μM and 250–15.6 nM, respectively. Nicotinamide (Sigma-Aldrich) was dissolved in ddH_2_O and diluted in media to 5 mM concentration. 5-azacytidine (Sigma-Aldrich) and 3-Deazaneplanocin A (Cayman Chemicals) were dissolved in DMSO and diluted in media to final concentration of 5 μM. CaCCInh-A01 (Sigma-Aldrich) was dissolved in DMSO and diluted in media to a final concentration of 10 μM. Every 48 h, media with fresh drug was changed during each experiment.

For short hairpin RNA (shRNA)-mediated knockdown, the PLKO.1 background vector was used (GE Life Sciences, Mickleton, NJ, USA). shRNA plasmids were packaged using a third-generation lentiviral system (pMDL, VSV-G and pRSV-REV), and expressed in HEK-293T cells. Human EPAS1 TRCN: 0000003805, 0000003807; Mouse EPAS1 TRCN: 0000082306, 0000082307. Mouse ANO1 TRCN: 0000173335, 0000193423. Scrambled shRNA was obtained from Addgene (Cambridge, MA, USA). Viral supernatant was collected 24 and 48 h post-transfection, and concentrated using 10-kDA Amicon Ultra-15 centrifugal filter units (Millipore, Billerica, MA, USA). For re-expression studies, human HIF-2α (ref. 18) open reading frame was sub-cloned into the pCDH-CMV-MCS-EF1-PURO mammalian expression vector.

### *In vivo* drug treatment

For *in vivo* drug studies, SAHA or DMSO was diluted daily in sterile 45% PEG/55% H_2_O (ref. 64), to the final concentration of 5 mg ml^−1^, and injected into the peritoneal cavity (50 mg kg^−1^ per day) once tumours reached 50–100 mm^3^. Mice were randomized to receive either treatment, and injected for up to 10 days before being killed 24 h after the last treatment.

### Mouse models

KPH2 mice were generated by crossing KP mice (*LSL-Kras*^G12D*/*+^*Trp53*^*f*l/fl^)^27^ and *Epas1*^fl/fl^ mice^33^, and KPA mice were generated by crossing KP mice and *Arnt*^*fl/fl*^ mice^65^. KP, KPH2 and KPA mice are on a mixed 129/C57BL/6 background. Tumours were generated in 8 weeks or older mix of male and female KP, KPH2 and KPA mice by injecting adenovirus expressing Cre-recombinase (Ad-Cre) into the right hind limb musculature as previously described^27^. KP, KPH2 and KPA mice were killed either 7 weeks after Ad-Cre injection, or when tumours reached the maximal permissible size. Mice were injected with BrdU (Sigma-Aldrich) 30 min before killing. For HT-1080 xenografts and KP250, KP and KPH2 allografts, 1 × 10^6^ cells were injected into both flanks of male Balb/c nu/nu mice (Charles River Laboratories, Burlington, MA, USA). To generate LPS246 xenografts, 2 × 10^6^ cells were injected into the flanks of scid/hairless (SHO) mice (Charles River Laboratories). Mice were injected with control tumours (scrambled shRNA) in one flank and experimental tumours (HIF-2α shRNA) in the other for LPS246 xenografts. Tumour size was measured every other day (except for SAHA studies, where tumours were measured daily), and animals were euthanized after 18–50 days post-tumour injection. Researchers were not blinded to the experimental groups during *in vivo* treatments. Animal well-being and comfort were monitored by certified veterinary staff. All mouse experiments were performed according to National Institutes of Health guidelines and approved by the University of Pennsylvania Institutional Animal Care and Use Committee. At the time the studies performed on KP, KPH2 and KPA animals were concluded, the University of Pennsylvania's Animal Care and Use Committee's guidelines on tumour load assays specified that implanted or autocthonous tumours in aggregate should not exceed 10% of the animal's body weight. All mice weighed >25 g each, and tumour weights for both the KPH2 and KPA cohorts were recorded as 0.2–2.2 g (that is, <10% body mass). To accurately measure tumour mass, the weights of contralateral control legs for each mouse were compared with legs-bearing tumours (Fig. 1b,d). Sample size for each experiment was estimated using the formula *n=*((z*α*_/2_ σ)/E)^2^, with *α*=0.05, and σ and E based off of initial HT-1080 xenograft experiments with scrambled and HIF-2α shRNA. No inclusion/exclusion criteria parameters were used in our studies. All tumours and animals used in experiments were included in data analysis.

### Tumour cell isolation

KP and KPH2 tumour cells were isolated from primary KP and KPH2 UPS tumours, respectively. Briefly, UPS were harvested from mice, and transferred to a cell-culture hood and cut into tiny pieces on a petri dish. Tumours were digested using collagenase (StemCell #07902, Vancouver, Canada) for 1 h at 37 °C. DMEM/F-12 (Sigma-Aldrich) with 2% foetal bovine serum was then added, and the digested mixture was filtered through a 40-μ filter. Red blood cells were lysed with ACK lysis buffer (Lonza, Allendale, NJ) according to the manufacturer's protocol. Cells were pelleted down, and washed with DMEM/F-12. Cells were cultured in DMEM (Cellgro, Manassas, VA) supplemented with 10% foetal bovine serum and incubated at 37 °C with 5% CO_2_ in a humidified cell-culture incubator. Cells were cultured at least four passages before being used for experiments, and *Epas1* recombination status was detected through PCR.

### Immunoblots

Protein lysates were prepared in RIPA lysis buffer, and separated on 10% SDS–polyacrylamide gel electrophoresis gels by electrophoresis. Subsequently, the lysates were transferred to nitrocellulose membranes and probed with the following antibodies and concentrations: HIF-2α 1:1,000 (Novus #NB100-122), HIF-1α 1:1,000 (Cayman Chemicals #1006421), caspase-3 1:1,000 (Cell Signaling #9662 Danvers, MA, USA), GAPDH 1:2,000 (Cell Signaling #2118), β-tubulin 1:1,500 (Cell Signaling #2146), phospho-4E-BP1 1:1,000 (S65, Cell Signaling #9451), 4E-BP1 1:1,000 (Cell Signaling #9452), phospho-S6K1 1:1,000 (T389, Cell Signaling #9205), c-Myc 1:5,000 (Abcam #32072), S6K1 1:1,000 (Cell Signaling #2708), phospho-AKT 1:1,000 (S473, Cell Signaling #9271), AKT 1:1,000 (Cell Signaling #9272), phospho-EGFR 1:1,000 (Y1068, Cell Signaling #3777), EGFR 1:1,000 (Cell Signaling #4267) ANO1 1:500 (Abcam ab64085), phospho-CAMKIIα 1:1,000 (T286, Cell Signaling #12716) and CAMKII 1:1,000 (Cell Signaling #11945). Uncropped immunoblot images are included in Supplementary Fig. 8.

### CT imaging

Computer tomography (CT) images were generated using an Imtek MicroCAT II microCT scanner. The settings for the scan were 80 kVp, 500 uA with a 375 ms exposure time per projection. 360 projections were taken for the scan lasting ∼6 min. Reconstruction was performed with the software provided with the scanner: MicroCAT: Image Reconstruction, Visualization, & Analysis. It used a Feldkamp Reconstruction with a Shepp-Logan Filter. Voxel size is 103 × 103 × 103 μm^3^. Tumour volume was measured from scans using Amide software, and measurements were confirmed by a board-certified radiologist who was blinded to the treatment used for each mouse.

### Immunohistochemistry and immunofluorescence

Immunohistochemistry and immunofluorescence were performed on 5-μm paraffin embedded tissue sections. For immunohistochemistry, enzymatic Avidin-Biotin Complex-diaminobenzidine staining (Vector Labs) was used with haematoxylin used for counterstaining nuclei according to standard protocols. Immunohistochemistry was performed on soft tissue sarcoma and normal arterial and skeletal muscle tissue array (US Biomax, Rockville, MD, USA #SO801a). The following primary antibodies and concentrations were used for immunohistochemistry: anti-BrdU 1:40 (Abcam #ab6326), anti-cleaved caspase-3 Asp 175 1:300 (Cell Signaling #9661) anti-Ki67 1:100 (Abcam #ab15580), anti-phospho-S6 (S235/236, Cell Signaling #4858) and anti-HIF-2α (ThermoPierce #PA1-16510). The following primary antibodies and concentrations were used for immunofluorescence: anti-CD31 1:50 (Abcam #ab28364), anti-pimonidazole FITC 1:100 (Hypoxyproble HP2). Mounting media with DAPI (Life Technologies #P36935) was applied last. Sections were imaged using a Leica DMRB microscope and an Olympus DP72 camera.

### Quantitative reverse trascriptase-PCR

Total RNA was isolated from cells using the TRIzol reagent protocol (Invitrogen) and from tumours and skeletal muscle tissue using the RNAeasy minikit (Qiagen). RNA was reverse transcribed using the High-Capacity RNA-to-cDNA kit (Applied Biosystems), and transcript expression was determined by quantitative PCR using the Applied Biosystems Viia7 system. Target cDNA levels were measured with Taq-Man primer/probe sets (Applied Biosystems) for the following mouse and/or human targets: *EPAS1* (human: HS01026142_M1, mouse: MM01236108_M1), *HIF1A* (human: HS00153153_M1, mouse: MM01283758), *SERPINE1* (human: HS01126606_M1, mouse: MM00435860_M1), *MYC* (mouse: MM00487804_M1), *CCND1* (mouse: MM00432359_M1), *CCND2* (mouse: MM00438070_M1), *MCM5* (mouse: MM01243769_M1), *CDKN1A* (mouse: MM00432448_M1), *HPRT* (human: HS01003268_G1, mouse: MM01318743_M1) and *ACTB* (human: HS01060665_G1, mouse: MM00607939_S1). Expression levels were normalized to *HPRT* and *ACTB*.

### Proliferation assays

Cells were plated in triplicate for each data point, and incubated overnight in tissue culture dishes. The following day, DMSO or SAHA, diluted in growth media, was added to the cells, and the cells were place either in normoxic conditions (21% O_2_) or hypoxic conditions (1% O_2_) using a Ruskinn InVivO_2_ 400 workstation. Media with drug was changed every 2 days. Cells were trypsinized, resuspended in PBS and counted using a haemocytometer on the days indicated.

### Chromatin immunoprecipitation

ChIP assays were performed using QuickChIP assay kits (Novus Biologicals). Briefly, five million KP250 cells were fixed in 1% formaldehyde for 10 min at 37 °C, and the reaction stopped by glycine. Cells were washed, collected and lysed using SDS buffer containing protease inhibitor cocktail, and then sonicated into DNA fragments of 300–800 bp in size. Supernatants were diluted and pre-cleared with salmon sperm DNA/protein A/G agarose, and a 10-μl aliquot was saved for input control. The rest of the sample was immunoprecipitated with 5 ug of the following antibodies overnight: Rabbit IgG (Cell Signaling), Histone H3 (Abcam), Histone H3 Acetyl K9+K14+K18+K23+K27 (Abcam). Antibody-nucleoprotein complexes were recovered by incubating 60 μl of salmon sperm DNA/protein A/G agarose for 1 h at 4 °C with rotation. Beads were washed and antibody-nucleoprotein complexes were eluted from protein A/G agarose beads in elution buffer, de-cross-linked by adding 20 μl 5 M NaCl and incubated at 65 °C. Subsequently, RNAase A and proteinase K were added and DNA fragments were purified using QIAquick PCR purification kit (Qiagen). Semi-quantitative PCR was performed with the following primers: Epas1 Forward: 5′-CATTACTCAGTCCTGCGCTAACTG-3′; Epas1 Reverse: 5′-CTCAGGACACTGCCGAGGATTGTA-3′; Hif1a Forward: 5′-AATCACTTGGAGACTTCCCTTGTT-3′; Hif1a Reverse: 5′CACGTTGCTCTCAGCCAATCAGGA-3′.

### Oncomine study design

We used publically available databases through Oncomine Research Premium edition software (version 4.5, Life Technologies) to query HIF-2α expression and survival data in sarcomas relevant to our study. For expression data, we evaluated the Barretina *et al.* (GEO accession number GSE21124) and Gobble *et al.* data sets (GEO accession number GSE30929)^34^,^35^, and for survival data the Gobble *et al.* data set was analysed.

### Survival analysis

Available patient survival data were obtained from Gobble *et al.* data set for liposarcoma patients through Oncomine. Patients were divided into two groups: tumours expressing the highest 50% of *EPAS1* ‘High *EPAS1*' and lowest 50% ‘Low *EPAS1'*, and Kaplan–Meier analyses were performed for overall survival of patients.

### RNA-seq

RNA was extracted from KP and KPH2 FFPE tumour sections using RNeasy FFPE kit (Qiagen #73504). Library preps and RNA-seq were performed by the Next Generation Sequencing Core at the University of Pennsylvania, using TruSeq Stranded Kit (Illumina #RS-122-2201), Ribo-Zero Gold Kit (Epicentre #MRZG126) and an HiSeq 100SR (Illumina). Raw sequence and quality files were aligned to the mouse genome (GRCm38) using the RNA alignment tool STAR (version 2.4, https://code.google.com/p/rna-star/). Unique transcript alignments were counted for each sample using htseq-count (version 0.6.1, http://www-huber.embl.de/users/anders/HTSeq/doc/count.html) against the RefSeq transcripts for GRCm38, yielding counts for 26,667 transcripts for each sample. Principle Components Analysis of raw count data revealed that one HIF-2α knockout sample was a technical outlier. This sample was excluded from statistical analysis.

### Differential expression and gene set enrichment analysis

Transcript count files for 3 HIF-2α knockout samples and four wild-type samples were analysed with DESeq2 (version 1.6, http://bioconductor.org/packages/release/bioc/html/DESeq2.html) for differential expression. Log2-transformed, normalized intensities were also exported from DESeq2 for visualization and used as input for Gene Set Enrichment Analysis (see below). Overall sample relatedness was visualized with Principal Components Analysis (PCA, as implemented in Partek Genomics Suite v6.6, Partek, Inc. St Louis, MO) using the normalized expression intensities for all transcripts. Log2-transformed expression intensities from these samples were imported into GSEA (version 2.1, http://www.broadinstitute.org/gsea/index.jsp). Enrichment of gene sets corresponding to Canonical Pathways (C2-CP from the Molecular Signatures Database v4.0, http://www.broadinstitute.org/gsea/msigdb/index.jsp) was tested with default settings. Tables of enriched pathways for genes up in HIF-2α knockout or wild type were exported, with a false discovery rate (FDR)<0.1 cutoff.

### TCGA bioinformatics analysis

Level 3 data comprising regions with altered copy number and methylation status in sarcoma were downloaded through the TCGA portal (https://tcga-data.nci.nih.gov/tcga/tcgaHome2.jsp) on 17 December 2014. Data were analysed with Partek software (version 6.6). Tumour samples for copy number analysis consisted of 260 primary tumours, 3 recurrent tumours and 1 metastatic tumour. The results from blood and normal controls were eliminated from the analysis as they contained no amplifications or deletions. Briefly, sample files were lined up and concatenated. Thresholds for amplifications and deletions were set at 0.55 and −0.4, as based on previously published literature. Methylation analysis was performed on 242 primary tumours, 2 recurrent tumours, 1 metastatic tumour and 4 normal tissues. Probes annotated for proximity to EPAS1 were evaluated. All probes were tested for differential beta values between normal and all tumour samples with analysis of variance (ANOVA) followed by Benjamini–Hochberg FDR correction.

### Ingenuity pathway analysis

DESeq2 statistical results for differential expression were uploaded to IPA. Genes with at least a 0.75 log_2_ ratio (±1.7 fold) and a FDR corrected *P*-value of <0.05 were analysed. The changes in the subset of these genes that were known to interact with mTOR were used to predict the activity changes of MTOR in KPH2 versus KP tumours. Canonical pathway analysis was also performed, with the top 15 most statistically significant pathways shown (orange bars, pathway predicted to be more active in KPH2 tumours; blue bars, pathway predicted to be less active in KPH2 tumours; grey bar, no activity pattern available).

### Statistical analysis

Statistical analysis was performed using Prism (GraphPad Software). Data are shown as mean+s.e.m. unless otherwise specified. Data were reported as biological replicates, with technical replicates indicated in figure legends. Unpaired two-tailed Student *t*-tests were performed to determine if results were statistically significantly different, with a *P*-value cutoff <0.05 considered significant. Variation within each data group was measured with Graphpad Prism software, and found to be similar between groups tested.

## Additional information

**Accession codes:** The RNA-seq data have been deposited in the Gene Expression Omnibus database under accession code: GSE67672.

**How to cite this article:** Nakazawa, M. S. *et al.* Epigenetic re-expression of HIF-2α suppresses soft tissue sarcoma growth. *Nat. Commun.* 7:10539 doi: 10.1038/ncomms10539 (2016).

## Supplementary Material

Supplementary Information Supplementary Figures 1-8 and Supplementary Table 1.
