# Supplementary material for: Epigenetic re-expression of HIF-2α suppresses soft tissue sarcoma growth
Source: Nat Commun. 2016 Feb 3;7:10539. doi: 10.1038/ncomms10539 (PMC4742834; doi:10.1038/ncomms10539)
Supplement: Supplementary Information — Supplementary Figures 1-8 and Supplementary Table 1. [file ncomms10539-s1.pdf]

EPIGENETIC RE-EXPRESSION OF HIF-2α SUPPRESSES SOFT TISSUE SARCOMA GROWTH

Supplementary Figure 1.

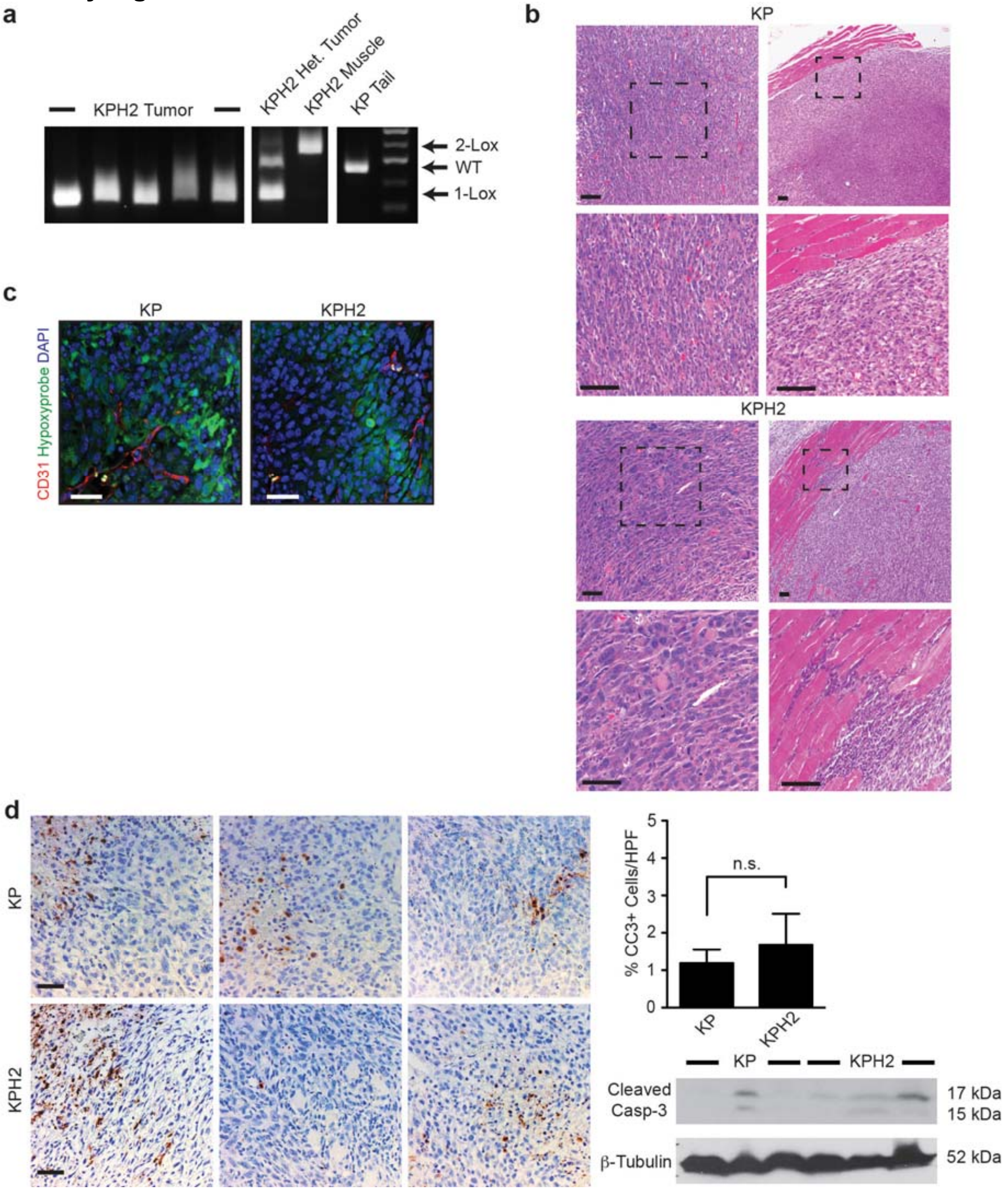

Supplementary Figure 1. Characterization of KP and KPH2 autochthonous UPS tumors.
a) Genotyping of KPH2 tumors shows efficient *Epas1* recombination. Controls include DNA derived from a KP tail (WT), muscle from a KPH2 mouse (2-lox), and a tumor from a *LSL-Kras*$^{G12D/+}$;*Trp53*$^{fl/fl}$; *Epas1*$^{fl/+}$ (KPH2 Het. Tumor) mouse. **b)** Left: Representative H&E stains of KP and KPH2 tumors at low magnification (upper) and higher magnification (lower). Histopathology of these UPS tumors were similar to prior reports using the KP mouse model[27]. Right: Local muscle invasion was observed, however it was more pronounced in KPH2 than KP tumors. Scale bar = 100 µm. **c)** KP and KPH2 tumors display areas of significant hypoxia, as demonstrated by immunofluorescence staining of Hypoxyprobe (green). CD31^{+} cells (red) and DAPI nuclear staining (blue) are shown. Scale bar = 50 µm. **d)** Left: Immunohistochemistry of cleaved caspase-3 (CC3) in KP and KPH2 tumors. Representative images are shown, and quantification of CC3^{+} cells from KP (*n* = 5) and KPH2 (*n* = 5) tumors is shown on the right. Right: Immunoblot of KP and KPH2 tumors for cleaved caspase-3. Scale bar = 50 µm. All data shown are the mean ± SEM.

Supplementary Figure 2

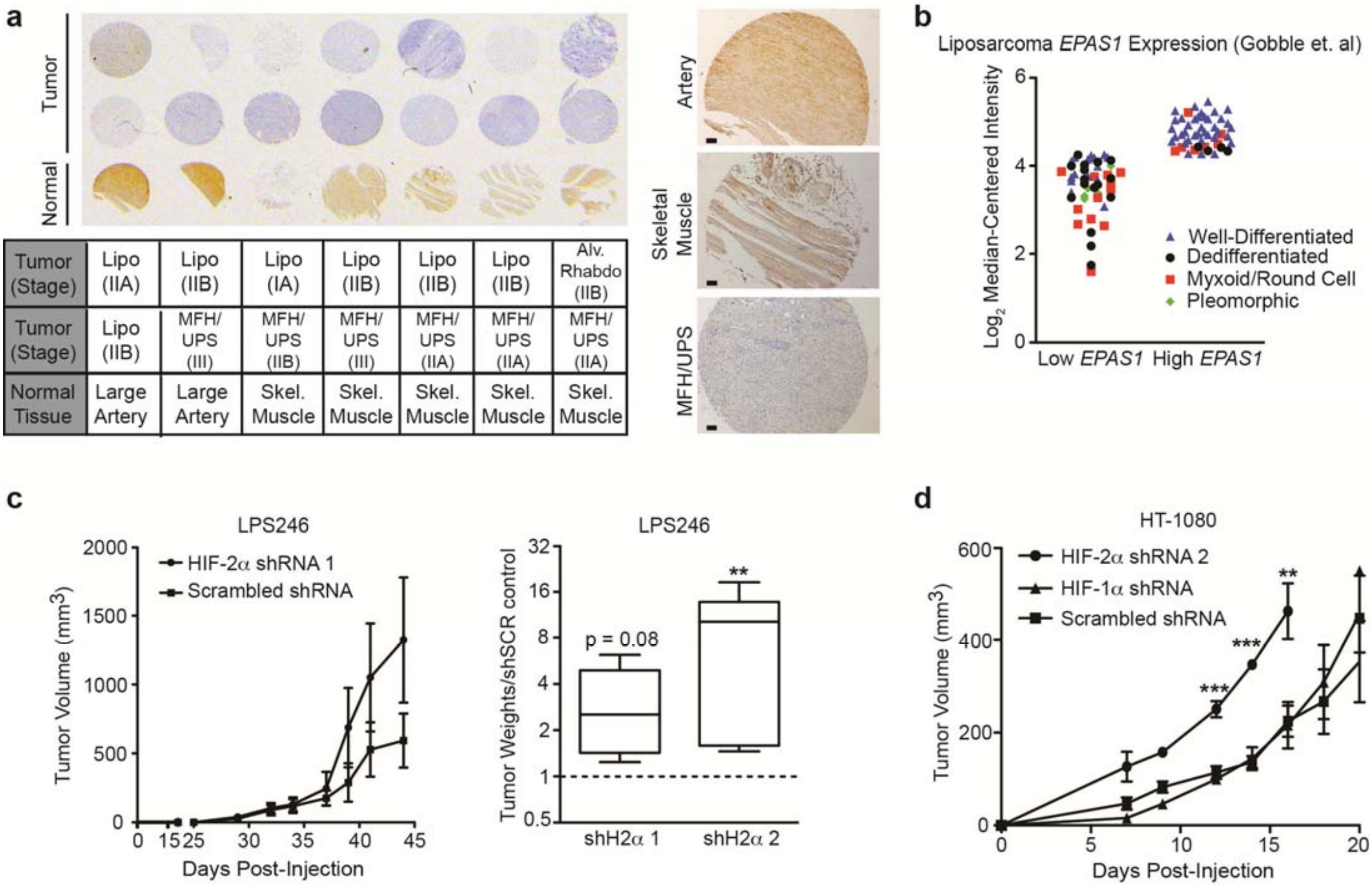

Tumor (Stage)	Lipo (IIA)	Lipo (IIB)	Lipo (IA)	Lipo (IIB)	Lipo (IIB)	Lipo (IIB)	Alv. Rhabdo (IIB)
Tumor (Stage)	Lipo (IIB)	MFH/ UPS (III)	MFH/ UPS (IIB)	MFH/ UPS (III)	MFH/ UPS (IIA)	MFH/ UPS (IIA)	MFH/ UPS (IIA)
Normal Tissue	Large Artery	Large Artery	Skel. Muscle	Skel. Muscle	Skel. Muscle	Skel. Muscle	Skel. Muscle

Supplementary Figure 2. HIF-2α inhibits tumor growth in other STS.
a) Left: IHC staining of HIF-2α was performed on a tissue array containing normal arterial (Large Artery) and skeletal muscle (Skel. Muscle) tissue, and liposarcoma (Lipo), alveolar rhabdomyosarcoma (Alv. Rhabdo), and MFH/UPS tissues. Histopathological characterization of each tissue sample is shown below. Right: Higher magnification of the indicated tissues in the array. Scale bar = 100 μm. **b)** *EPAS1* expression of individual tumor samples from "Low *EPAS1*" and "High *EPAS1*" group, with each tumor's subtype indicated in the legend. **c)** Left: Tumor volume of LPS246 xenografts with scrambled (n = 5) or HIF-2α shRNA 1 (n = 5). HIF-2α knockdown increased LPS246 tumor volume, although results did not reach statistical significance (p = 0.10). Right: Ratio of the indicated HIF-2α shRNA's tumor weight to scrambled shRNA control (shSCR) tumor weight in each mouse. **d)** Tumor volume of HT-1080 xenografts with scrambled shRNA (n = 10), HIF-1α shRNA (n = 10), and HIF-2α shRNA 2 (n = 10). ** = $P < 0.01$. *** = $P < 0.001$. All P values were calculated using a two-tailed Student's t-test. All data shown are the mean ± SEM.

Supplementary Figure 3

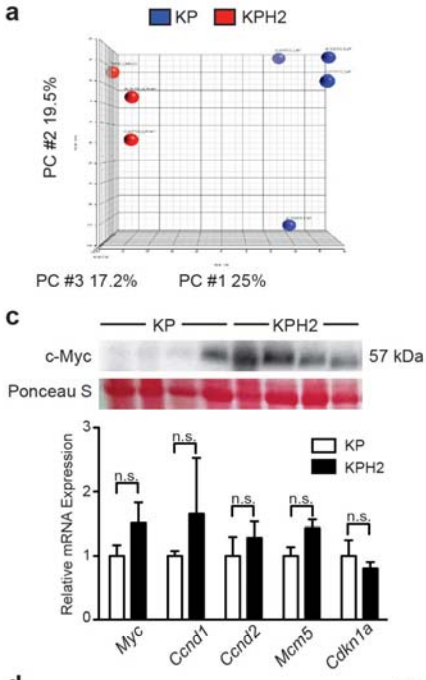

b Pathways elevated in KPH2 tumors

NAME	Enrichment Score	NOM p-val	FDR q-val
REACTOME_RNA_POL_I_PROMOTER_OPENING	0.8356897	0	0
REACTOME_RNA_POL_I_TRANSCRIPTION	0.77312213	0	0
REACTOME_MUSCLE_CONTRACTION	0.8028224	0	0
REACTOME_STRIATED_MUSCLE_CONTRACTION	0.8733387	0	0
REACTOME_RNA_POL_I_RNA_POL_III_AND_MITOCHONDRIAL_TRANSCRIPTION	0.70115525	0	0
REACTOME_PACKAGING_OF_TELOMERE_ENDS	0.8177489	0	0
REACTOME_MEIOTIC_RECOMBINATION	0.7329803	0	0
REACTOME_DEPOSITION_OF_NEW_CENPA_CONTAINING_NUCLEOSOMES_AT_THE_CENTROMERE	0.7739824	0	0
REACTOME_MEIOTIC_SYNAPSIS	0.7425791	0	0
REACTOME_MEIOSIS	0.67534465	0	0
REACTOME_TELOMERE_MAINTENANCE	0.7211001	0	3.42E-04
REACTOME_CHROMOSOME_MAINTENANCE	0.61956173	0	0.00633
REACTOME_MYOGENESIS	0.7753517	0	0.013298
REACTOME_TRANSCRIPTION	0.56643796	0	0.01669681
KEGG_TIGHT_JUNCTION	0.5862549	0	0.02276062

Pathways elevated in KP tumors

NAME	Enrichment Score	NOM p-val	FDR q-val
KEGG_COMPLEMENT_AND_COAGULATION_CASCADES	-0.6790581	0	0.01086171
REACTOME_LIGAND_GATED_ION_CHANNEL_TRANSPORT	-0.7953941	0	0.00757279
KEGG_ASTHMA	-0.82444316	0	0.04852867
KEGG_CYTOKINE_CYTOKINE_RECEPTOR_INTERACTION	-0.50743854	0	0.05043586
REACTOME_ORGANIC_CATION_ANION_ZWITTERION_TRANSPORT	-0.838359	0	0.09813815
REACTOME_DEGRADATION_OF_THE_EXTRACELLULAR_MATRIX	-0.6972977	0	0.0893279

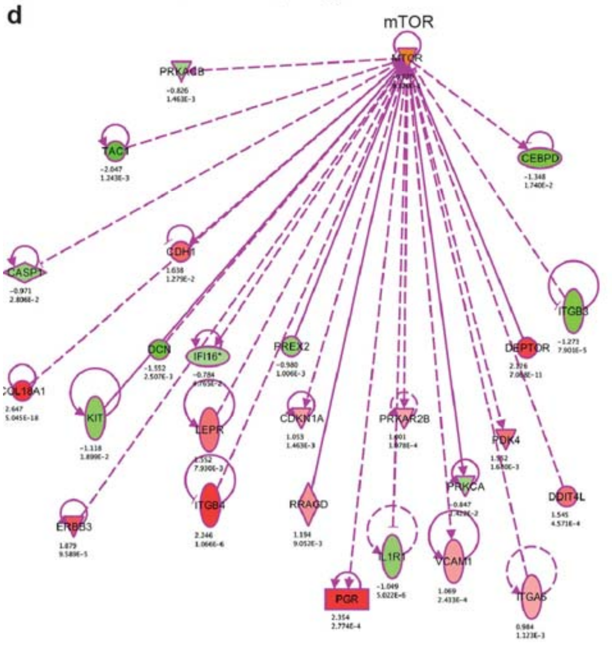

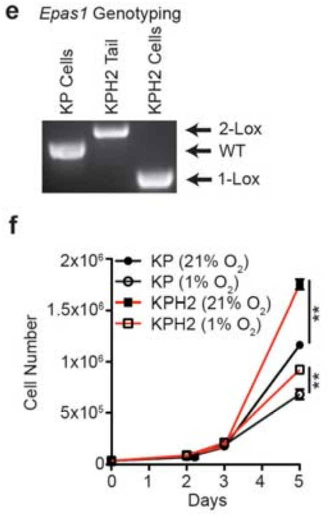

Supplementary Figure 3. Analysis of RNA-seq data from KP and KPH2 tumors.
a) Principle component analysis (PCA) mapping of RNA-seq data from KP (*n* = 3) and KPH2 (*n* = 4) tumors.
b) List of the top gene set enrichment analysis (GSEA) categories elevated in KPH2 and KP tumors, with false discovery rate (FDR) < 0.1. Enrichment score and nominal p-value for each category are also displayed. **c) Top:** ImmunoblotqPCR of c-Myc in KP and KPH2 derived UPS tumors, with Ponceau S as loading control. **Bottom**: qRT-PCR of several downstream Myc targets (*Myc*, *Ccnd1*, *Ccnd2*, *Mcm5*, *Cdkn1a*) showed no statistically significantly different expression levels in KP (*n* = 4) and KPH2 (*n* = 3) tumors. **d)** Ingenuity Pathway Analysis (IPA) prediction of mTOR activity in KPH2 versus KP tumors, based upon mRNA expression changes from RNA-seq. Shown are interactors with mTOR, as calculated by IPA software, predicting mTOR to be activated. Green = decreased mRNA expression in KPH2 tumors. Red = increased mRNA expression in KPH2 tumors. Orange = increased predicted activity. **e)** PCR of KP and KPH2 tumor derived cell lines demonstrating efficient floxing of the *Epas1* allele in the KPH2 cell line. DNA from a KPH2 tail is shown as a positive control for 2-lox. **f)** Proliferation of KP and KPH2 tumor derived under 21% O_2 or 1% O_2. Each line represents three independent experiments performed in duplicate. ** = *P* < 0.01. All *P* values were calculated using a two-tailed Student's *t*-test. All data shown are the mean ± SEM.

Supplementary Figure 4

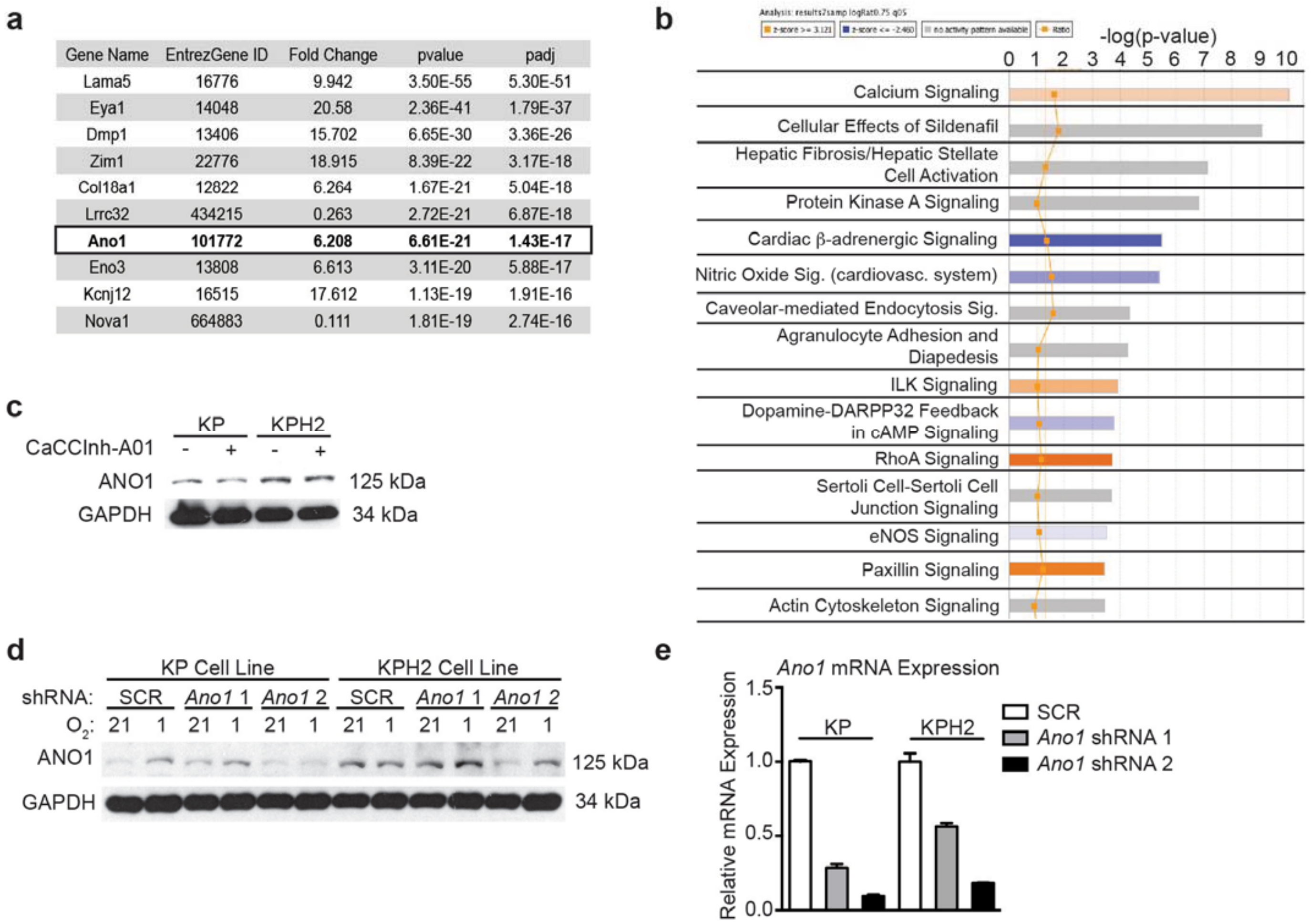

Gene Name	EntrezGene ID	Fold Change	pvalue	padj
Lama5	16776	9.942	3.50E-55	5.30E-51
Eya1	14048	20.58	2.36E-41	1.79E-37
Dmp1	13406	15.702	6.65E-30	3.36E-26
Zim1	22776	18.915	8.39E-22	3.17E-18
Col18a1	12822	6.264	1.67E-21	5.04E-18
Lrrc32	434215	0.263	2.72E-21	6.87E-18
**Ano1**	**101772**	**6.208**	**6.61E-21**	**1.43E-17**
Eno3	13808	6.613	3.11E-20	5.88E-17
Kcnj12	16515	17.612	1.13E-19	1.91E-16
Nova1	664883	0.111	1.81E-19	2.74E-16

Supplementary Figure 4. *Ano1* is upregulated in HIF-2α deficient UPS tumors
a) Ten most differentially expressed genes from RNA-seq of KP and KPH2 tumors. Fold changes indicated are expression changes in KPH2 tumors compared to KP tumors. **b)** IPA analysis of biological processes differentially expressed between KP (n=4) and KPH2 (n=3) tumors. Data was generated from mRNA changes from RNA-seq. Processes predicted by IPA analysis to be more active in KPH2 tumors are colored orange, processes predicted to be less active are colored blue, and those with no predictive pattern available are colored grey. The statistical significance of each biological process being differentially expressed is shown on the x-axis. **c)** Ano1 protein levels in KP and KPH2 tumor derived cells *in vitro*, representative of 2 independent experiments. **d)** Immunoblot for Ano1 in KP and KPH2-derived cell lines infected with scrambled or 2 independent *Ano1* shRNAs. **e)** *Ano1* expression of KP and KPH2-derived cell lines with scrambled (SCR) or *Ano1* shRNAs. *Ano1* shRNA 2 had a more robust knockdown of *Ano1* expression. Each bar represents two independent experiments performed in triplicate. All *P* values were calculated using a two-tailed Student's *t*-test. All data shown are the mean ± SEM.

Supplementary Figure 5

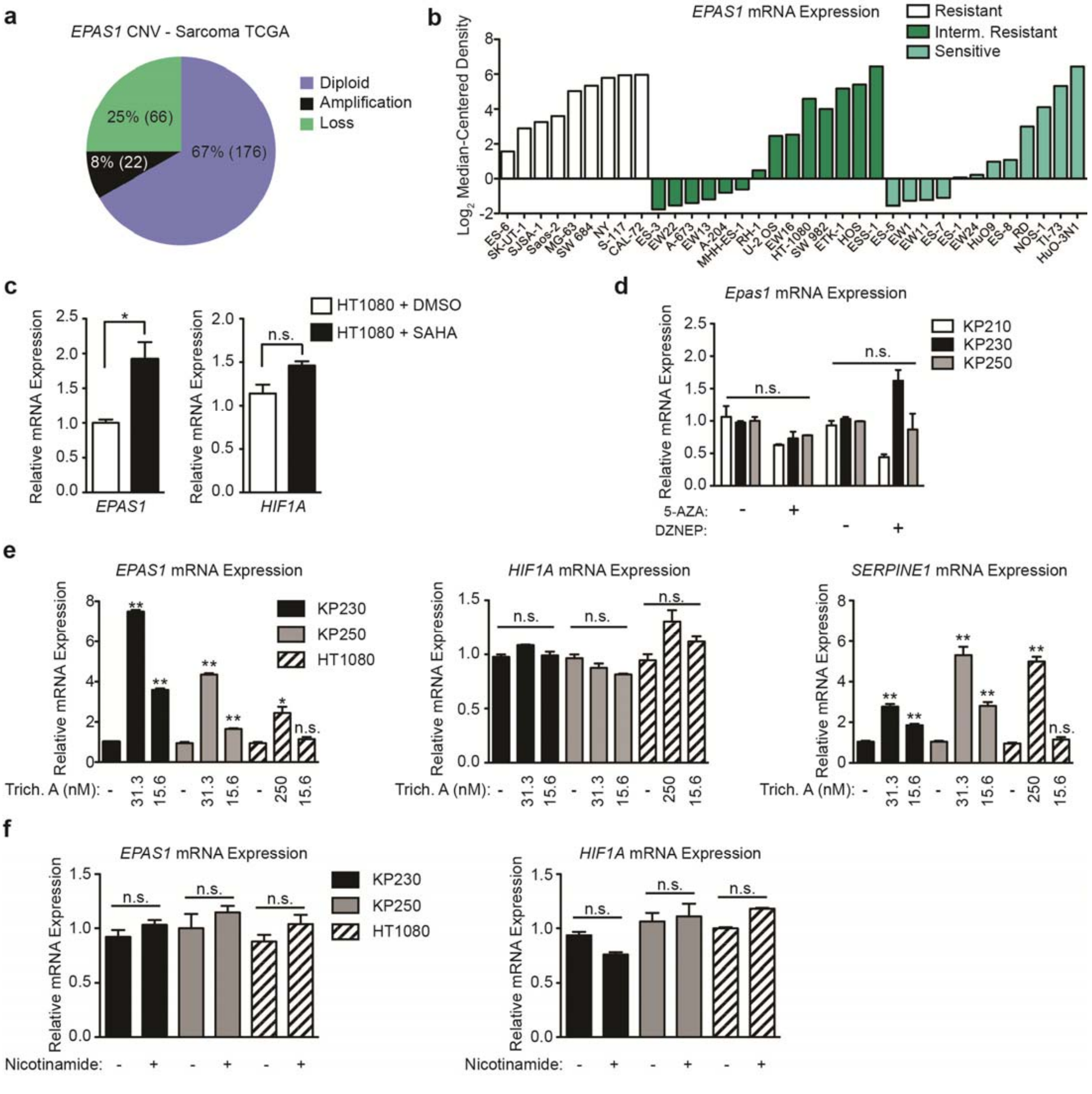

Supplementary Figure 5. CNV of *EPAS1* in sarcoma patients, and effects of epigenetic drugs on STS.
a) Copy number variation (CNV) of all sarcoma samples in the TCGA database (*n* = 264) across the *EPAS1* locus, grouped into diploid, copy number amplification, and copy number loss. **b)** *EPAS1* mRNA levels of each sarcoma cell line and their sensitivity to vorinostat treatment. **c)** *EPAS1* and *HIF1A* mRNA expression of HT-1080 cells upon DMSO control and SAHA (2 µM) treatment. Bars represent two independent experiments performed in triplicate. * = $P < 0.05$. **d)** *Epas1* mRNA levels in three independently derived UPS cell lines after treatment with methylation inhibitor 5-azacytidine (5-AZA, 5 µM) and EZH2 inhibitor 3-Deazaneplanocin A (DZNEP, 5 µM). Bars represent two independent experiments performed in triplicate **e)** *EPAS1*, *HIF1A*, and *SERPINE1* mRNA expression changes upon HDAC inhibitor Trichostatin A (Trich. A) treatment in KP230, KP250, and HT-1080 cell lines at the indicated concentrations. Bars represent two independent experiments performed in triplicate. DMSO served as control treatment. * = $P < 0.05$. ** = $P < 0.01$. **f)** Effect of nicotinamide treatment (5 mM) on KP230, KP250, and HT-1080 cells on *EPAS1* and *HIF1A* mRNA. H_2O served as control treatment. Bars represent three independent experiments performed in triplicate. All *P* values were calculated using a two-tailed Student's *t*-test. All data shown are the mean ± SEM.

Supplementary Figure 6

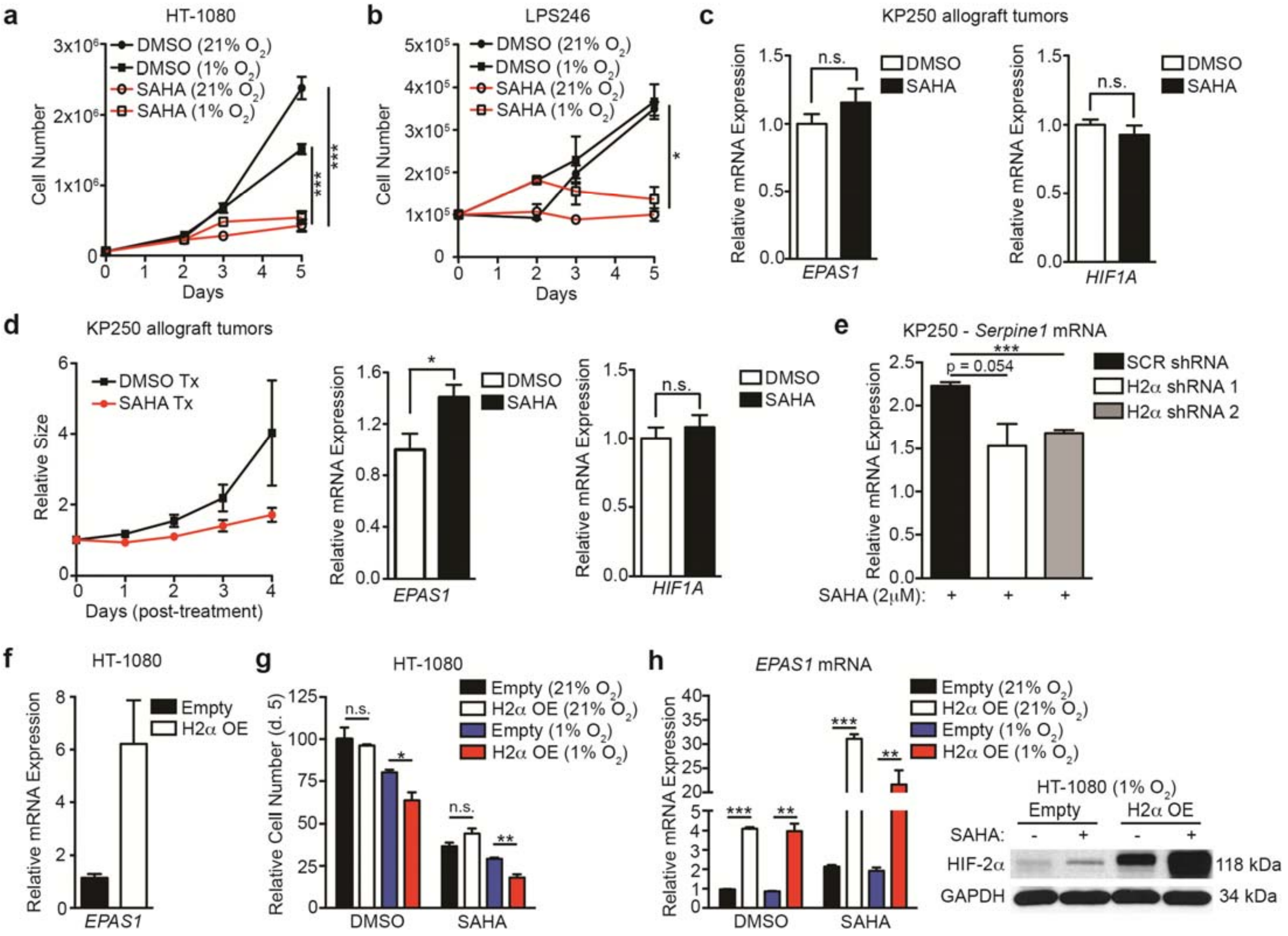

Supplementary Figure 6. Resistance to HIF-2α re-expression develops to SAHA monotherapy *in vivo*.
a) – b) Cell proliferation of **a)** HT-1080 and **b)** LPS246 cells treated with DMSO control or SAHA (2 μM) in 21% O_2 and 1% O_2 conditions. Drug treatment began on day 1. * = $P < 0.05$. *** = $P < 0.001$. Bars represent biological triplicates. **c)** *Epas1* and *Hif1a* mRNA expression of KP250 allograft tumors isolated from mice treated with 8 days of DMSO ($n = 5$) or SAHA ($n = 5$). **d)** Analysis of KP250 allografts treated with a short 4 day course of SAHA. Left: Relative tumor size increase of KP250 allografts treated with DMSO control ($n = 4$) or SAHA (50 mg/kg/day, $n = 4$). Right: *Epas1* and *Hif1a* mRNA expression from KP250 allografts after 4 days of DMSO or SAHA treatment. * = $P < 0.05$. **e)** mRNA expression of the HIF-2α target gene *Serpine1* in KP250 cells with scrambled (SCR) shRNA, and two independent HIF-2α (H2α) shRNAs, treated with SAHA (2 μM) *in vitro*. Expression is relative to KP250 SCR shRNA treated with DMSO. Bars represent at least two independent experiments performed in triplicate (Error bars are ± SEM). **f)** mRNA expression of *EPAS1* in HT-1080 cells with empty vector control (Empty) or HIF-2α expressing construct (H2α OE). Bars represent two independent experiments performed in triplicate. **g)** Relative cell proliferation of HT-1080 with empty vector control or HIF-2α overexpression, grown under normoxic (21% O_2) or hypoxic (1% O_2) conditions for 5 days in the presence of DMSO or SAHA (750 nm). Values are normalized to the number of HT-1080 empty vector cells grown under 21% O_2 and DMSO. Each bar represents biological triplicates. * = $P < 0.05$. ** = $P < 0.01$. **h)** Left: mRNA expression of *EPAS1* in HT-1080 cells with empty vector control of HIF-2α overexpression grown in conditions described in part **g**. Each bar represents biological triplicates. Right: Immunoblot of HIF-2α in HT-1080 cells overexpressing HIF-2α, grown under hypoxic (1% O_2) conditions in the presence of DMSO or SAHA (750 nM) for 5 days. ** = $P < 0.01$. *** = $P < 0.001$. All P values were calculated using a two-tailed Student's *t*-test. All data shown are the mean ± SEM.

Supplementary Figure 7

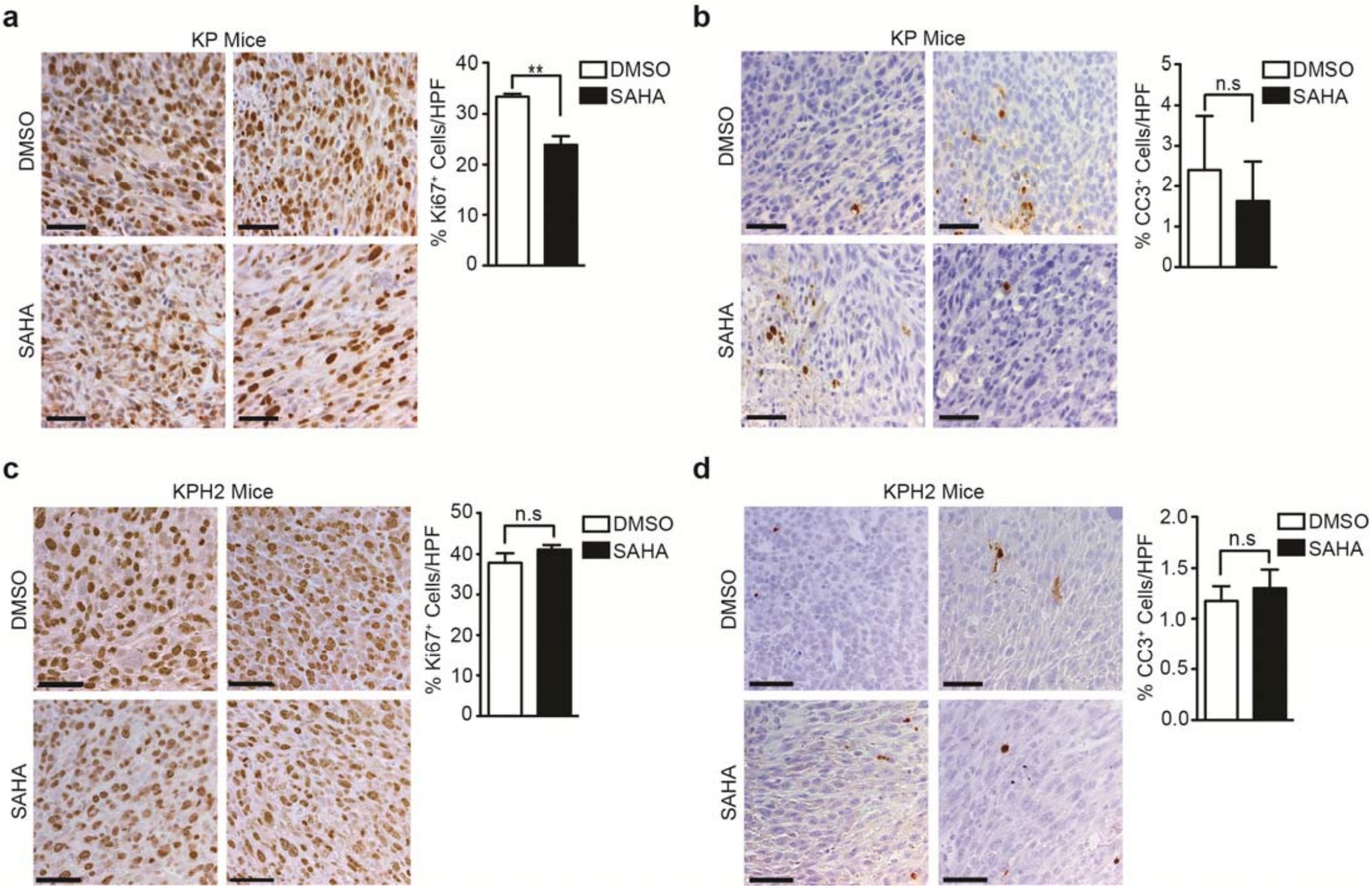

Supplementary Figure 7. SAHA decreases proliferation in UPS autochthonous tumors in a HIF-2α dependent manner. **a) - b)** Immunohistochemical staining of autochthonous KP tumors treated with DMSO (n = 5) or SAHA (50 mg/kg/day, n = 5). **a)** Representative images of Ki67 stained tumor sections (left), and quantification of the percentage of Ki67$^+$ cells per high-powered field in DMSO treated or SAHA treated tumors (right). **b)** Representative images of cleaved caspase-3 (CC3) stained tumor sections (left), and quantification of the percentage of CC3$^+$ cells in DMSO treated or SAHA treated tumors (right). ** = P < 0.01. **c) - d)** Immunohistochemical staining of autochthonous KPH2 tumors treated with DMSO (n = 4) or SAHA (50 mg/kg/day, n = 5). Representative images of **c)** Ki67 and **d)** cleaved caspase-3 (CC3) stained tumors sections, with quantification of percentage of Ki67$^+$ and CC3$^+$ cells per high power field. Scale bars (**a-d**) = 50 μM. All P values were calculated using a two-tailed Student's t-test. All data shown are mean ± SEM.

Supplementary Figure 8

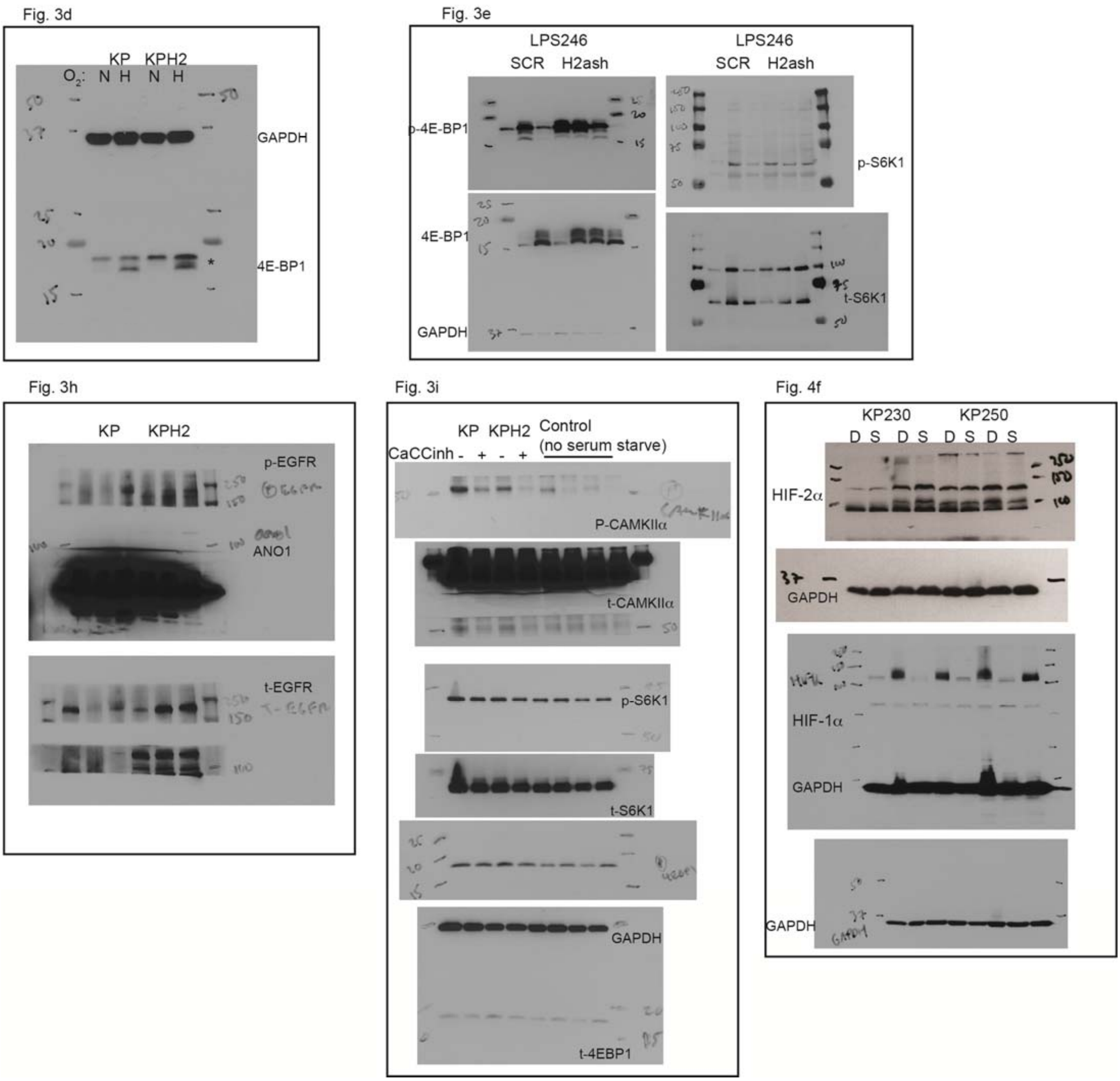

Supplementary Figure 8. Uncropped immunoblots, corresponding to the indicated figures in the manuscript.

Supplementary Table 1.

Column #	Probeset ID	Gene Symbol	Chrom.	Genomic Coordinate	p-value	stepup (p-value)	Primary Solid Tumor - Mean (242)	Recurrent Solid Tumor - Mean (2)	Metastatic (1)	Solid Tissue Normal - Mean (4)
192556	cg10107473	EPAS1	2	46566094	0.130669	0.871675	0.424158	0.0800508	0.696835	0.417186
232422	cg12485219	EPAS1	2	46588351	0.247106	0.940267	0.873533	0.929329	0.847345	0.909147
320478	cg17518825	EPAS1	2	46523461	0.360529	0.982055	0.773478	0.915536	0.71872	0.714626
234440	cg12608132	EPAS1	2	46564242	0.433804	0.998912	0.776584	0.951892	0.86575	0.815803
119931	cg06093355	EPAS1	2	46547693	0.435278	0.998912	0.562781	0.312648	0.696904	0.445636
36451	cg01780482	EPAS1	2	46524612	0.466253	0.998912	0.0346882	0.0441955	0.0307917	0.0324421
397383	cg22488486	EPAS1	2	46612964	0.501649	0.998912	0.774084	0.858186	0.827826	0.860843
105209	cg05290658	EPAS1	2	46613024	0.509671	0.998912	0.709895	0.885029	0.722639	0.730558
379875	cg21276379	EPAS1	2	46523790	0.530208	0.998912	0.255735	0.43146	0.160772	0.29615
100712	cg05049980	EPAS1	2	46612798	0.552615	0.998912	0.895718	0.879227	0.927032	0.911881
199610	cg10507988	EPAS1	2	46593271	0.565311	0.998912	0.957028	0.972071	0.824108	0.982008
348703	cg19311375	EPAS1	2	46542361	0.587291	0.998912	0.289864	0.116312	0.303494	0.196377
382771	cg21494367	EPAS1	2	46612724	0.590392	0.998912	0.787722	0.912403	0.76663	0.810458
124940	cg06388946	EPAS1	2	46525020	0.625483	0.998912	0.059278	0.0655241	0.0416855	0.0547567
252876	cg13681847	EPAS1	2	46595230	0.666025	0.998912	0.802085	0.891575	0.797961	0.862216
153620	cg07901411	EPAS1	2	46527523	0.680617	0.998912	0.598231	0.569492	0.859313	0.585124
117177	cg05943554	EPAS1	2	46609618	0.706844	0.998912	0.916131	0.938724	0.907709	0.941333
440343	cg25124739	EPAS1	2	46527098	0.75321	0.998912	0.199266	0.0588295	0.188653	0.123225
452812	cg25941751	EPAS1	2	46613544	0.778893	0.998912	0.556207	0.632838	0.710377	0.651947
177473	cg09240515	EPAS1	2	46605834	0.783934	0.998912	0.739612	0.829867	0.687392	0.780589
429337	cg24460563	EPAS1	2	46525678	0.792873	0.998912	0.0722973	0.0501502	0.0426928	0.0454716
280894	cg15129144	EPAS1	2	46527958	0.810399	0.998912	0.832117	0.928815	0.862193	0.864142
369472	cg20623601	EPAS1	2	46526843	0.85669	0.998912	0.144665	0.0493967	0.129207	0.0744125
464583	cg26627511	EPAS1	2	46525649	0.88317	0.998912	0.0441569	0.0311959	0.034116	0.0241031
259317	cg13997788	EPAS1	2	46593223	0.88545	0.998912	0.808804	0.750741	0.844322	0.818448
90530	cg04518274	EPAS1	2	46525028	0.897986	0.998912	0.0381435	0.0437533	0.0194133	0.0385946
2308	cg00097800	EPAS1	2	46593258	0.903052	0.998912	0.922265	0.9456	0.862235	0.936751
6982	cg00330888	EPAS1	2	46524148	0.925201	0.998912	0.0205977	0.0161684	0.00973106	0.0130607
422783	cg24046888	EPAS1	2	46526785	0.947341	0.998912	0.181641	0.21014	0.135803	0.245746
27461	cg01313320	EPAS1	2	46526265	0.948711	0.998912	0.0583034	0.0420988	0.0398939	0.0384673
237156	cg12784625	EPAS1	2	46524557	0.971302	0.998912	0.0188266	0.0129969	0.0139515	0.0141106
216056	cg11501862	EPAS1	2	46524365	0.978321	0.998912	0.0209065	0.0151255	0.0143506	0.0166117
171175	cg08871383	EPAS1	2	46524363	0.993367	0.998912	0.01748	0.0144179	0.0105785	0.0151849
40194	cg01960526	EPAS1	2	46524359	0.995223	0.998921	0.0214806	0.0194334	0.0148275	0.0173618
175345	cg09119656	EPAS1	2	46556344	?	?	?	?	?	?
201302	cg10600644	EPAS1	2	46530198	?	?	?	?	?	?
283314	cg15291914	EPAS1	2	46526566	?	?	?	?	?	?
313292	cg17091312	EPAS1	2	46558320	?	?	?	?	?	?

Supplementary Table 1. DNA methylation across the *EPAS1* locus from The Cancer Genome Atlas (TCGA) sarcoma samples. Primary solid tumors (*n* = 242), recurrent solid tumor (*n* = 2), metastatic (*n* = 1), and normal solid tissue (*n* = 4) were analyzed, and the mean Beta-values for each group shown. P-values comparing tumor tissue verses normal tissue Beta-values were calculated for each probe was calculated using DEseq.
